# Cell-specific rates of sulfate reduction and fermentation in the sub-seafloor biosphere

**DOI:** 10.3389/fmicb.2023.1198664

**Published:** 2023-07-24

**Authors:** Marion Jaussi, Bo Barker Jørgensen, Kasper U. Kjeldsen, Bente A. Lomstein, Christof Pearce, Marit-Solveig Seidenkantz, Hans Røy

**Affiliations:** ^1^Department of Biology, Aarhus University, Aarhus, Denmark; ^2^Department of Geoscience, Aarhus University, Aarhus, Denmark

**Keywords:** cell-specific carbon oxidation rates, basal power requirement, sulfate reducing microorganisms, fermentative microorganisms, deep biosphere

## Abstract

Microorganisms in subsurface sediments live from recalcitrant organic matter deposited thousands or millions of years ago. Their catabolic activities are low, but the deep biosphere is of global importance due to its volume. The stability of deeply buried sediments provides a natural laboratory where prokaryotic communities that live in steady state with their environments can be studied over long time scales. We tested if a balance is established between the flow of energy, the microbial community size, and the basal power requirement needed to maintain cells in sediments buried meters below the sea floor. We measured rates of carbon oxidation by sulfate reduction and counted the microbial cells throughout ten carefully selected sediment cores with ages from years to millions of years. The rates of carbon oxidation were converted to power (J s^−1^ i.e., Watt) using the Gibbs free energy of the anaerobic oxidation of complex organic carbon. We separated energy dissipation by fermentation from sulfate reduction. Similarly, we separated the community into sulfate reducers and non-sulfate reducers based on the *dsrB* gene, so that sulfate reduction could be related to sulfate reducers. We found that the per-cell sulfate reduction rate was stable near 10^−2^ fmol C cell^−1^ day^−1^ right below the zone of bioturbation and did not decrease with increasing depth and sediment age. The corresponding power dissipation rate was 10^−17^ W sulfate-reducing cell^−1^. The cell-specific power dissipation of sulfate reducers in old sediments was similar to the slowest growing anaerobic cultures. The energy from mineralization of organic matter that was not dissipated by sulfate reduction was distributed evenly to all cells that did not possess the *dsrB* gene, i.e., cells operationally defined as fermenting. In contrast to sulfate reducers, the fermenting cells had decreasing catabolism as the sediment aged. A vast difference in power requirement between fermenters and sulfate reducers caused the microbial community in old sediments to consist of a minute fraction of sulfate reducers and a vast majority of fermenters.

## 1. Introduction

As continued deposition gradually buries marine sediments, they become increasingly isolated from the surface world. Dissolved electron acceptors can be supplied to the isolated microbial community from above, but the pool of organic carbon that fuels microbial life is mostly constrained to the stationary solid phase (e.g., Komada et al., 2013). Thus, a microbial community must live from the finite amount of organic carbon buried with it in the sediment. The anaerobic food chain is inefficient, and repeated cycles of cell death and reassimilation of necromass would lead to rapid loss of carbon (Orsi et al., 2020). Nevertheless, we find microorganisms in ancient sediments that are still thriving and are slowly degrading the old and refractory organic matter (Røy et al., 2012). This implies that the rates of mineralization are exceedingly low. Indeed, the reactivity of organic matter decreases steeply in aging sediment (Middelburg, 1989; Boudreau and Ruddick, 1991; Shang, 2023).

As the rates of carbon mineralization decrease with increasing age, so does the size of the microbial community (Røy et al., 2012). Although the assembly of the deep biosphere community conserves a part of the surface community (Starnawski et al., 2017), the decreasing community size with increasing age and depth in the sediment is not merely due to a slow death of the surface community. This can be seen by the continuous production of dead microbial cells (necromass) far in excess of the size of the original community (Lomstein et al., 2012), and by the fact that the estimated biomass turnover times of the sedimentary microbes is much shorter than the age of the sediment they live in Biddle et al. (2006), Hoehler and Jørgensen (2013), Braun et al. (2017). Thus, the microbial community in the deep sediment column must largely be in steady state with respect to their basal power requirement and the local availability of energy at any time. As the rate of liberation of labile carbon substrates from the refractory organic matter decreases with time, the size of the microbial community is decreasing accordingly. If the energy supply were in excess, the community would grow and thereby reduce the energy flux available per cell to approach the basal power requirement. If the energy supply falls below the basal power requirement, some cells will die, and this increases the per-cell energy availability (LaRowe and Amend, 2015b). Thus, we expect that the ever-decreasing energy turnover in aging marine sediments will force cells in the deep biosphere to constantly exist at the lowest power dissipation that will sustain their community (Hoehler and Jørgensen, 2013; Lever et al., 2015).

The lower limit, i.e., the basal power requirement, of prokaryotic cells is, most likely, set by physical and chemical decay processes in the cells such as the rate of leakage of membrane potential, the rate of depurination of nucleic acids, and the rate of racemization of amino acids. None of these processes are, however, constrained well enough to confidently calculate the basal power requirement of the individual cells (Lever et al., 2015). Yet, the intrinsic rate of racemization of aspartic acid, which is the amino acid with the highest rate of racemization, indicate that this process leads to the largest unavoidable loss of energy (Brinton et al., 2002; Onstott et al., 2014). Indeed, a gene encoding the enzyme (Protein-L-iso aspartate(D-aspartate) O-methyltransferase), which recognizes damaged L-isoapartyl and D-aspartyl residues in proteins and catalyzes their repair while still within the protein, was found widely distributed and expressed in deeply buried sediments in the Baltic Sea (Mhatre et al., 2019).

The lowest basal power requirement for prokaryotes in the deep biosphere is difficult to determine in the laboratory yet experiments with axenic bacterial cultures maintained without addition of substrates for prolonged time under so-called long-term stationary phase have shown general mechanisms of adaptation in cell respiration to extreme nutrient limitation (e.g., Riedel et al., 2013; Robador et al., 2019). To avoid artifacts related to laboratory cultivation, we have searched for the basal power requirement of cells in the natural environment by relating the catabolic rate of a community to the community size (Hoehler and Jørgensen, 2013) directly in sediments of varying age, ranging from tens to millions of years old. To limit the number of variables in the data, we focused on the sulfatic zone (Canfield and Thamdrup, 2009; Jørgensen, 2021), where anaerobic respiration is dominated by sulfate reduction. The goal of our study was to identify if, and how, the availability of energy controlled the microbial community size and the per-cell metabolic rate in the energy-starved deep biosphere. In addition, we compared the community size of the two main metabolic guilds, fermenters and sulfate reducers, living syntrophically in sulfate-rich sediments, with the power available to each of the guilds.

## 2. Materials and methods

### 2.1. Sampling sites

Gravity-cores from eight different geographic locations were retrieved for this study during 2012–2014. When possible, the gravity-cores were supplemented with either box-cores or Rumohr cores from the same site. Two additional datasets were retrieved from the databases of the Ocean Drilling Program (ODP) Leg 201 and Integrated Ocean Drilling Program (IODP) Leg 323. The goal of the site selection was to access samples from an extensive range of well-constrained sediment ages within the sulfatic sediment zone. In addition, the sedimentary and geochemical settings were selected such that the rate of dissimilatory sulfate reduction could be determined with good accuracy. The sampling sites can be found in Table 1, in Supplementary Figure S1, and at https://drive.google.com/open?id=1rYE3drQ6eSkWIjRFQtpxwL265bM&usp=sharing.

**Table 1:** Coordinates of the coring sites and general description of the cores.

Site	Drilling hole/core name	Water depth (m)	Latitude	Longitude	Temp. (°C)*	Length of core (m)	Age (year)*^§^*
Little Belt	SKA14-05-B25	38	55°00.258′ N	10°06.519′E	9	0.035	20
Greenland Glacier Fjord	SA13-ST8-47G	475	64°40.7078′ N	50°17.4672′ W	2	5.67	200
Greenland Main Fjord	SA13-ST5-30G	622	64°25.3479′ N	51°30.6209′ W	2	6.06	466
Greenland Side Fjord	SA13-ST6-40G, SA13-ST6-35R	389	64^°^29.0604′ N	50°42.3240′ W	2	5.61	4,600
Iceland Basin	DA12-11-1-GC01	2120	61°37.04′ N	20°43.26′ W	3	4.20	12,500
Greenland Continental Shelf	SA13-ST3-20G, SA13-ST3-17R	498	64°26.742′ N	52°47.6478′ W	4	5.92	12,600
Faroe Bank	DA12-11-ST2-GC03	742	60°46.94′ N	009°47.62’W	8	5.89	55,100
South Azores	DA14-ST1-GC01	2515	37°17.773 N	27°04.934 W	3.2	5.96	300,000
Bering Sea	IPDP Exp. 323, Hole U1342-B	818	54° 49.7004′ N	176° 55.0232′ E	2–6	43	1,000,000
Eastern Equatorial Pacific	ODP Leg 201, Hole 1,226-B	3297	3°5.24′S	90°49.12′ W	1.7–25.6	417	15,600,000

*Bottom water temperature (or temperature gradient down through the sediment for Eastern Equatorial Pacific and Bering Sea long cores). § approximated age at the bottom of the cores.

### 2.2. Subsampling of sediment cores

Gravity-cores were retrieved specifically for the study and the greatest care was taken to avoid oxygen exposure and heating. Thus, the cores were sectioned into 1-meter sections immediately after core recovery and the full core sections were capped and placed horizontally near *in situ* temperature. Rumohr cores were capped with overlying water and stored vertically until processing. All cores were taken at high latitudes, which helped to avoid elevated temperatures in the upper water column and on deck.

Extraction of pore water and solid-phase sampling were completed within 4–48 h after sediment recovery. Pore-water was extracted with Rhizon soil-moisture samplers (Rhizosphere Research Products, Wageningen, Netherlands) through 4 mm holes drilled through the plastic core-liners every 10–25 cm within the top 1 m of the gravity-cores and Rumohr Lot cores. Below 1 m, the sample resolution in the gravity-cores was 20 to 25 cm. The first milliliter of extracted pore water was discarded, and the rest of the pore water was collected in evacuated Exetainers (Labco) before distribution for further analyses. Solid phase samples were collected with sterile plastic syringes with cut-off tip through windows cut into the core liner with a vibrating saw (Røy et al., 2014), after the outer sediment in contact with the liner had been removed. The solid-phase samples were taken from the same depths as the pore water and care was taken to only sample one side of the core (working half of the core). For selected cores, the archive-half was scanned by an ITRAX x-ray diffraction core scanner (Croudace et al., 2006).

### 2.3. Determination of age-models

The loss of sediment from the top of the gravity-cores, due to the core catcher and the violent penetration of the sediment surface, was estimated by matching the pore water profiles of NH_4_^+^, SO_4_^2−^, and δ^13^C_DIC_. The estimated sediment loss was then used to extend the age-models of the cores all the way to the sediment–water interface (see section 3.4 for analytical details). The matched pore water profiles can be found in the cited literature for each individual cores.

The average age of the upper 5 few cm of sediment in Little Belt core (SKA14-05-B25) was estimated based on correlation to IODP expedition 347 site M0059 (Andrén et al., 2015), which was retrieved at the same location.

The age of the rapidly accumulating sediment in the Glacier Fjord (core SA13-ST8-47G) was analyzed via short-lived natural gamma emitters (^210^Pb and ^226^Ra). The sedimentation rate was estimated from the least-squares fit to the natural log of excess ^210^Pb in the core and the output of a one-dimensional two-layer advection–diffusion model that accounted for both biomixing and compaction with depth (Lavelle et al., 1985; Kuzyk et al., 2015). The data and the procedures are described in detail in Pelikan et al. (2019).

Calcareous mollusk shells and organic worm-tubes were collected in the Greenland Continental Shelf (core SA13-ST3-20G), Main Fjord of Nuuk Kangerlua (also known as Nuuk Fjord or Godthåbsfjord; SA13-ST5-30G), Side Fjord Kapisillit Kangerluat of Nuup Kangerlua (SA13-ST6-40G), Iceland Basin (DA12-11-ST1-GC01), and Faroe Bank (DA12-11-ST2-GC03) sediment cores for ^14^C age determination by Accelerator Mass Spectrometry at Aarhus AMS Centre, Aarhus University. The ^14^C ages were calibrated using the Marine13 radiocarbon calibration curve (Reimer et al., 2013) with no further regional reservoir correction (ΔR = 0). The age models were reported as calibrated ^14^C years BP (Before Present, where present = AD 1950). For the analysis of the organic matter reactivity here, however, the ages were calculated to years before collection (AD 2013) to relate to mineralization age. The procedures and data are described in detail in Petro et al. (2019).

The age-depth model of the South Azores core (DA14-ST1-GC01) was developed from the sediment description and from calcium (Ca) and iron (Fe) measurements using an ITRAX x-ray fluorescence (XRF) core scanner (Croudace et al., 2006). The profile of Ca/Fe, which may here be considered a proxy for marine productivity, was aligned with marine isotope stages and the age of the stages was determined according to LR04 Benthic Stack (Lisiecki and Raymo, 2005).

The age-depth model of the Bering Sea site (IODP Exp323, Hole U1342B) was retrieved from Knudson and Ravelo (2015), who based their age model on calcite δ^18^O of benthic foraminifera, which they too correlated to the LR04 Benthic Stack (Lisiecki and Raymo, 2005). The depths were converted into ages using a linear interpolation between the measurements. Below 35 mbsf (meters below seafloor), there was a shift in stratigraphic unit (Unit II), where the age versus depth was not clearly resolved (Expedition 323 Scientists, 2011). Therefore, data below this depth were excluded from our study.

The age-depth model for the Eastern Equatorial Pacific core (ODP Leg 201, Hole 1,226-B) was based on one unique ^14^C measurement and six biostratigraphic boundaries from the Pleistocene to the Middle Miocene (Shipboard Scientific Party, 2003; D'Hondt et al., 2004), previously assessed at site 846 of ODP Leg 138 (Shipboard Scientific Party, 1992). The depths were converted into ages using a linear interpolation between the seven age points. Below 388 mbsf, the age model was interpolated linearly, based on a basement age of 16.5 million years at the base of the core.

### 2.4. Pore water analyses

#### 2.4.1. Ammonium

Aliquots of 1 mL pore water for analysis of dissolved ammonium were transferred to 2.5 mL Eppendorf tubes and frozen at −20°C until analysis. Ammonium concentrations were analyzed by spectrophotometry of the blue indophenols formed when ammonium is dissolved in a weak alkaline solution with salicylate, hypochlorite, and sodium nitroprusside (Bower and Holm-Hansen, 1980). After dilution of the pore water samples with MilliQ water (up to 50 times), 1 mL of the dilution was first mixed with 120 μL salicylic acid catalyst, then 200 μL of alkaline-hypochlorite solution (1 part of alkaline-citrate solution and 9 parts of 5% sodium hypochlorite) was added to the reaction tube. After incubating the reaction mix for 1 h, the absorbance was measured at 650 nm on a spectrophotometer (FLUOstar Omega, BMG Labtech GMBH, Ortenberg, Germany).

#### 2.4.2. Dissolved inorganic carbon

Pore-water for analysis of dissolved inorganic carbon (DIC) was transferred to glass vials (Zinsser) filled up to the brim (*ca.* 2 mL), closed without headspace and stored at 4°C until analysis. Subsamples were transferred to 10 mL helium-flushed exetainers and the DIC was transferred to the headspace as CO_2_ by reaction with phosphoric acid. The CO_2_ content of the headspace was then analyzed by gas chromatography using a GC-IRMS with helium as carrier gas (CTC Analytics GC-pal autosampler, Thermo scientific GasBench II, Thermo scientific ConFlo IV, Thermo scientific Finnigan Delta V plus IRMS). The carbon isotopic composition (δ^13^C) of the CO_2_ was determined relative to the VPDB standard using LSVEC (δ^13^C: -46.4‰ _VPDB_) and NBS 19 (δ^13^C: +1.95‰ _VPDB_) for calibration.

#### 2.4.3. Sulfate

Aliquots of 300 μL pore water for analysis of sulfate (SO_4_^2−^) were transferred to 2.5 mL Eppendorf tubes and ventilated at room temperature for 20 min (only samples with low H_2_S and high SO_4_^2−^) or flushed with humidified CO_2_ to remove hydrogen sulfide before storage at 4°C until analysis. The pore water was then diluted with MilliQ water (10–100 times), and the sulfate concentration was determined by ion chromatography (Dionex ICS 2500 with AS 18 column and ED 50 electrochemical detector). An initial KOH eluent concentration of 20 mmol L^−1^ was used for the analysis, followed by a column flush at 32 mmol L^−1^ (Røy et al., 2014). In samples from Greenlandic waters, the measured sulfate concentrations were normalized to the chloride concentration to correct for possible dilution and evaporation errors.

### 2.5. Solid phase analyses

#### 2.5.1. Porosity and density

Porosity and density of the sediment were determined using 2 cm^3^ of wet sediment and calculated from the weight loss of sediment after drying at 100°C until constant weight. Porosity was calculated from water content multiplied by wet density.

#### 2.5.2. Total organic carbon

Total organic carbon (TOC) was determined by combusting dry ball-mill-powdered sediment in an elemental analyzer [FLASH EA (1112 series), Thermo Scientific]. To remove the inorganic carbon, the sediment samples were pre-treated with 5% (w/w) sulfurous acid until the samples no longer produced CO_2_ bubbles (Braun et al., 2017). Once re-dried and homogenized, aliquots of 50 mg acidified sediment were packed into tin cups and burned in the elemental analyzer. The content of organic matter is given as nmol C cm^−3^ fresh wet sediment, calculated based on the carbon content, the porosity, and the dry density. By this volume-specific unit, the organic carbon content can be directly compared to its volume-specific rate of oxidation.

#### 2.5.3. Total cell abundance

Samples for total cell abundance determination were taken with 2.5 mL cut-off syringes. 1 mL of sediment was transferred into 4 mL sterile and saline paraformaldehyde solution (35 g L^−1^ NaCl and 2 g L^−1^ paraformaldehyde, final concentration of PFA in preserved samples 0.1%), mixed thoroughly and stored at 4°C until analysis. Microbial cells were quantified by fluorescence microscopy of cell extracts based on Kallmeyer et al. (2008) and Morono et al. (2013). The cell extraction consisted of a chemical detachment by a detergent mix (100 mM EDTA, 100 mM sodium pyrophosphate, and 1% (v/v) Tween 80) and methanol, followed by a mechanical detachment by three times 10 s sonication at 7 W. After each detachment treatment, the microbial cells were separated from sediment particles using density centrifugation with 50% Nycodenz (AXIS-SHIELD PoC AS). The cell extracts were pooled and filtrated on black polycarbonate membrane filters (25 mm, GTBP, 0.2 μm pore size) and stained with DAPI solution on the filter. A minimum of 400 cells were counted on at least 12 fields of view with an epifluorescence microscope (Axiovert 200 M Zeiss, Germany). An additional dissolution with acetic acid (Kallmeyer et al., 2008) was tested at all sites, but only applied to the calcareous Faroe Bank sediment.

#### 2.5.4. Relative abundance of sulfate reducers

The relative abundance of sulfate-reducer cells compared to total cell abundance was determined by quantitative PCR (qPCR) of *dsrB* and *16S* rRNA- genes in extracted DNA. Mud samples were taken in cut-off 5 mL syringes that were immediately frozen at −80°C. Total nucleic acids were extracted from 0.5 to 1.0 g thawed sediment with a combination of enzymatic and chemical pre-treatment and the FastDNA Spin Kit for Soil (MP Biomedicals) as described by Kjeldsen et al. (2007). The abundance of *dsrB-* and of bacterial and archaeal *16S* rRNA gene copies in the DNA extracts were quantified by SYBR green-based qPCR according to Jochum et al. (2017) and Starnawski et al. (2017). The *dsrB* gene encodes for the β-subunit of dissimilatory (bi)sulfite reductase, which is a diagnostic marker gene for sulfate-reducing prokaryotes. Triplicate reactions were performed for each DNA extract. For 2–3 samples from each core the qPCR assays were performed on ten-fold serial dilutions of the DNA extract to test for the presence of co-extracted PCR inhibitors (inhibition effects were not observed). The *dsrB* gene qPCR assay conditions and performance were reported previously (Jochum et al., 2017). The assay standard curves were linear within a range of 10^2^ to 10^8^ target gene copies μL^−1^ template, with an efficiency between 102 and 107% and with *R*^2^ > 0.99. The relative abundance of sulfate-reducing microorganisms in the total microbial community was calculated from *dsrB* gene copy numbers and the sum of bacterial and archaeal *16S* rRNA gene copy numbers assuming that sulfate reducers on average harbor 1 *dsrB* copy per genome (Jochum et al., 2017) while bacterial and archaeal cells on average harbor 4 and 2 *16S* rRNA gene copies, respectively (Suna et al., 2013). Based on analysis of DNA extraction negative control samples and using 2 μL DNA template, the limit of quantification of the *dsrB* assay was approx. 7400 *dsrB* gene copies per gram of wet sediment. The limit of quantification in gene copies cm^−3^ wet sediment varied from site to site depending on porosity and density, but was always close to 10^4^. See supplementary information for primer sequences and further details.

### 2.6. Volume-specific carbon oxidation rates

#### 2.6.1. Sulfate reduction rates by ^35^SO_4_^2−^ tracer incubation

Sulfate reduction rates (SRR) were measured experimentally by ^35^SO_4_^2−^ tracer incubation in cut-off 5 mL syringes according to Røy et al. (2014). Ten μL of carrier free ^35^SO_4_^2−^ tracer (100 to 250 kBq) was injected in the center of each sediment sample. The samples were incubated at *in situ* temperature for 6 to 24 h in the dark, under anoxic conditions in air-tight plastic bags with an oxygen scrubber inside (Oxoid™ AnaeroGen™, Thermo Scientific). The incubations were stopped by freezing the mini-cores at −20°C or − 80°C. Later, the samples were distilled to separate the total reduced inorganic sulfur (TRIS) from sulfate by a cold chromium distillation (Kallmeyer et al., 2004, including modifications as recommended by Røy et al., 2014). The total radioactivity of the sediment before distillation and the distilled fraction of total reducible inorganic sulfur (TRIS) were measured separately by liquid scintillation counting (Packard Tri-Carb 2,900 TR). Killed blank samples that consisted of sediment transferred to ZnAc (20% w/v) before tracer injection were used to test the procedure background and limits of quantification. The sulfate reduction rates (SRR) were calculated according to Jørgensen (1978a).


(1) $$\mathrm{SRR}=\left[\mathrm{sulfate}\right]\times \varphi \times \left(\frac{a_{\mathrm{TRIS}}}{a_{\mathrm{TOT}}}\right)\times 1.06\times t^{-1}\;$$

where [sulfate] is the pore-water sulfate concentration, φ is the porosity, a_TRIS_ the radioactivity of TRIS, a_TOT_ the sediment radioactivity before distillation, t the incubation time, 1.06 a correction factor for the estimated isotope discrimination against ^35^SO_4_^2−^ (Jørgensen and Fenchel, 1974).

The measured rates of dissimilatory sulfate reduction were related to the oxidation of complex organic matter with the net-formula C_27_H_28_O_7_ (LaRowe and Amend, 2015a). This implies a nominal oxidation state of carbon of −0.52 and a C:S ratio of 1.77:1 according to the stoichiometry:


(2) $$\begin{array}{l} C_{27}H_{28}O_{7}+13\;H_{2}O+15.25\;{\mathrm{SO}}_{4}^{2-}\to \\ 27\;{\mathrm{HCO}}_{3}^{-}+15.25\;{\mathrm{HS}}^{-}+11.75\;H^{+} \end{array}$$

Sulfate reduction rates can be measured with ^35^SO_4_^2−^ tracer down to 0.02 pmol SO_4_^2−^ cm^−3^ day^−1^, especially if the concentration of sulfate is low (Glombitza et al., 2016). We refrained from measurements in old and sulfide-free sediments to avoid reoxidation of reduced radiotracer, avoid contaminating with atmospheric oxygen in samples with poor redox-buffer, and avoid slow drift away from *in situ* conditions during long incubations. Thus, the incubation times could be held shorter than 24 h and the injected activity of ^35^SO_4_^2−^ tracer could be held below 250 kBq per sample.

#### 2.6.2. Carbon oxidation rates based on modeling of NH_4_^+^ profiles

Complete mineralization of organic matter under sulfate-reducing conditions releases DIC and NH_4_^+^ in the same ratio as the C:N ratio of the organic matter that is being mineralized. But DIC is involved in precipitation and dissolution of carbonates, and the net rate of DIC production does not always correspond to the rate of carbon mineralization. We therefore used the NH_4_^+^ production rates to indirectly determine the carbon oxidation rates with greater accuracy in older sediments far from the sediment–water interface, where the rates were too low for determination with ^35^SO_4_^2−^ but the long diffusive distances increased the sensitivity of reaction–diffusion modeling. Ammonium production rates were determined by fitting a reaction–diffusion model to NH_4_^+^concentration profiles, assuming steady state and one-dimensionality (Boudreau, 1996) using the software PROFILE (Berg et al., 1998). The program solves the mass balance of NH_4_^+^ in the pore water:


(3) $$R_{NH_{4}^{+}}=-\frac{d}{\mathrm{dz}}\left(\varphi \times \left(D_{s}\right)\;\frac{\mathrm{dC}}{\mathrm{dz}}\right)\;$$

where R_NH4+_ is the rate of production of ammonium in the pore water, z is the sediment depth in meters below seafloor, φ the porosity of the sediment, D_s_ the diffusion coefficient of ammonium in the sediment, C the pore water ammonium concentration. The boundary conditions of the model were based on the measured concentrations of NH_4_^+^ at the upper and lower boundaries; exceptions are mentioned in Supplementary Table S1. The diffusion coefficient of ammonium in the sediment (D_s_) was determined using the relation:


(4) $$D_{s}=\frac{D_{0}}{\left(1+3\times \left(1-\varphi \right)\right)}\;$$

where D_0_ is the diffusion coefficient in seawater corrected for salinity and temperature (Boudreau, 1996).

##### 2.6.2.1. Calculation of diffusion coefficients in sediment with low porosity

For the site 1226-B in the Eastern Equatorial Pacific, the D_s_ for NH_4_^+^ was calculated from formation factors (FF), because this approach is more appropriate than Eq. 4 in very compact sediment, and because this approach has been used by previous authors who worked on data from the same site (Wang, 2006). The FF were interpolated from the measurements by a locally weighted least squares fit (*loess function*, in the R Stats Package (R Core Team, 2013), smoothing parameter =0.25). At the bottom of the core (374–418 mbsf), where FF measurements were not available, these factors were determined according to the empirical equation of Archie’s law:


(5) $$\mathrm{FF}=\varphi^{-1.8812}\times 10^{0.1916}$$

(Wang, 2006). FF was used to calculate the tortuosity (τ^2^) according to Boudreau (1996):


(6) $$\tau^{2}=\left(\mathrm{FF}\times \varphi \right)\;$$

The sediment diffusion coefficient (D_s_) was then calculated by dividing the temperature-corrected molecular diffusion coefficient of ammonium in pore water (D_0_) by the tortuosity.


(7) $$D_{s}=\frac{D_{0}}{\tau^{2}}$$

##### 2.6.2.2. Temperature-correction of diffusion coefficients In thermal gradients

The software PROFILE does not directly allow the diffusion coefficient (D_0_) to vary with depth, which is necessary in deep cores due to the geothermal gradient. To accommodate this deficiency, we calculated the difference between the diffusion coefficients calculated by PROFILE according to Eq. 4, and the temperature-corrected D_s_ at each depth. This difference was fed into the model as D_b_. At runtime, PROFILE will add D_s_ and D_b_, resulting in a correct diffusion coefficient (Wehrmann et al., 2011).

The reaction zones solved by PROFILE were fixed to a minimum of three zones when the model allowed. Data from the top and bottom reaction zones were rejected due to poor sensitivity, unconstrained transport coefficients, and strong influence of the boundary conditions, leaving a central reaction zone with high confidence in the calculated ammonium production rates.

##### 2.6.2.3. Calculation of carbon oxidation from NH_4_^+^ production

The ratio between DIC and NH_4_^+^ production rates in pore water can be calculated from the ratio between the concentrations of DIC and NH_4_^+^ without calculating the actual fluxes (Jørgensen and Parkes, 2010). The procedure is less sensitive to noise in the DIC data than reaction–diffusion modeling, it is independent of porosity-effects on diffusion coefficients, and it allowed us to transform our ammonium production rates (section 3.6.2.2) into the equivalent DIC production rates. Thus, the DIC: NH_4_^+^ production rate for each site was found as the slope of so-called parameter plots of NH_4_^+^ vs. DIC concentrations in the pore water, multiplied by the ratio of D_0_ of HCO_3_^−^ and D_0_ of NH_4_^+^ to account for the fact that NH_4_^+^ diffuses away faster than DIC. As the precipitation of calcium carbonate influences the DIC concentration, and therefore the apparent C:N ratio of mineralized organic matter, we determined the DIC: NH_4_^+^ production rate only in non-carbonate sediments. As all calculated DIC: NH_4_^+^ ratios fell in a narrow range we used the median value from all stations to transform the volume-specific rates of NH_4_^+^ production to volume-specific rates of carbon oxidation.

### 2.7. Reactivity of organic matter

The reactivity of the sedimentary organic matter was assessed in each sediment sample from the momentary first-order rate constant of its decay (k, in y^−1^):


(8) $$C_{\mathrm{ox}\;\mathrm{rate}}=k\times \mathrm{TOC}$$

(9) $$k=\frac{C_{\mathrm{ox}\;\mathrm{rate}}\;}{\mathrm{TOC}}$$

where C_ox rate_ is the rate of organic carbon mineralization based on SRR or NH_4_^+^ production rates (nmol C cm^−3^ y^−1^) and TOC is the measured concentration of total organic carbon (nmol C cm^−3^) at the same depth. The relation between k and sediment age was then derived by fitting a non-biased linear model to log–log transformed data and transposing this relation back to linear space according to Flury et al. (2016).

### 2.8. Mean cell-specific carbon oxidation rates

The mean cell-specific carbon oxidation rates were calculated by dividing the volume-specific carbon oxidation rates by the total cell abundance at the same depth. Similarly, the mean cell-specific sulfate reduction rates were assessed by dividing the volume-specific sulfate reduction rates by the number of sulfate-reducing cells. The latter was estimated by multiplying the relative abundance of *dsrB* genes (in %) by the total cell abundance at each depth.

### 2.9. Thermodynamic calculations

Complex sedimentary organic matter was represented by the net-formula C_27_H_28_O_7_ (LaRowe and Amend, 2015a), which has a nominal oxidation state of carbon (NOSC) of −0.52.

Since the standard free energy of organic matter is closely linked to the mean oxidation state of carbon, we can assess ΔG^0^_ox_ of the half reaction from C_27_H_28_O_7_ to CO_2_ per carbon atom via the relationship presented by LaRowe and Van Cappellen (2011) at 25°C and 1 bar:


(10) $$\Delta G_{\mathrm{ox}}^{0}=60.3-28.5\times \mathrm{NOSC}$$

We summed ΔG^0^_ox_ and the standard free energy of the sulfate reduction half-reaction (ΔG^0^_red_) calculated from tabulated Gibbs energy of formation for HS^−^, SO_4_^2−^ and H^+^ (Kulik and Harff, 1993). The total Gibbs energy of the reaction (ΔG_r_) was then calculated from ΔG^0^ and the pore water concentrations of HS^−^, SO_4_^2−^, HCO_3_^−^ and H^+^:


(11) $$\Delta G_{r}=\Delta G^{0}+R\times T\times \ln\underset{i}{\prod }a_{i}{}^{\nu_{i}}$$

where *R* is the gas constant (0.00831 kJ mol ^−1^ K^−1^), and T is the temperature (K).

$\underset{i}{\prod }a_{i}{}^{\nu_{i}}=\left(\frac{\prod_{i}a_{\left(product\right)}{}^{\nu_{i}}}{\;\prod_{i}a_{\left(substrate\right)}{}^{\nu_{i}}}\right)$ is the mass action expression where *a_i_* is the activity of the i^th^ component of the reaction, and *vi* is its stoichiometric ratio.

We used activity coefficients: 0.172 for SO_4_^2−^, 0.6592 for HS^−^, 0.6843 for HCO_3_^−^, retrieved from (Kulik and Harff, 1993) at an ionic strength of 0.647. Organic matter as a solid has an activity of 1 regardless of its concentration. We assumed a pH of 7, which influenced the activity of HCO_3_^−^ based on DIC concentration and HS^−^ based on total H_2_S concentration. For simplicity, the calculation was done at 25°C (298 K).

Likewise, ΔG_r_ of acetate (CH_3_COO^−^) oxidation to CO_2_ with sulfate as electron acceptor was calculated from Eq. 12, using the following stoichiometry:


(12) $${\mathrm{CH}}_{3}{\mathrm{COO}}^{-}+{\mathrm{SO}}_{4}^{2-}\to 2{\mathrm{HCO}}_{3}^{-}+{\mathrm{HS}}^{-}$$

For acetate, we used the same activity coefficient as HCO_3_^−^ and assumed pH = 7. Acetate concentrations in the Greenland cores were available from Glombitza et al. (2015), but such data are rare because acetate is difficult to measure in saline water at the relevant concentrations (Glombitza et al., 2014). But the most complete datasets indicate that the acetate concentrations in cold marine sediments are remarkedly constant due to thermodynamic constraints (Glombitza et al., 2019; Beulig et al., 2022). We therefore use the mean value of the acetate concentration in the Greenlandic cores (5.96 μM ± 1.6) in the calculation of ΔG_r_ for the Eastern Equatorial Pacific sediments where no measurements were available. To estimate the Gibbs energy liberated by fermentation, we subtracted ΔG_r_ from oxidation of acetate to CO_2_ via sulfate reduction from the total ΔG_r_ from C_27_H_28_O_7_ all the way to CO_2_.

### 2.10. ODP and IODP data

The data from ODP Leg 201, site 1226, in the Eastern Equatorial Pacific were retrieved from the Janus Web Database.^1^ Pore-water NH_4_^+^ concentration and porosity were obtained from Hole 1226-B and the porosity measurements were interpolated to the same depth resolution as NH_4_^+^. Cell counts from Hole 1226-B were retrieved from Parkes et al. (2005), who used direct counts with acridine orange, fluorescent dye, without cell extraction. TOC data (weight %) were retrieved from Janus web database and recalculated to nmol cm^−3^. Note that most TOC concentrations were measured in hole 1226-E.

Data from IODP expedition 323, site U1342, in the Bering Sea were retrieved from the Janus database and from published studies. The depth-matching between parameters (porosity from Hole C, pore water from Hole B) was done via the corrected composite depth scale (CCSF-A) in meter core composite depth below seafloor. This depth scale was constructed by Expedition 323 Scientists (2011) based on multiple drillings holes, A–D, and was calculated by adding a specific cumulative offset to the CSF-A depth scale of each core. We used ammonium concentration as our reference sample resolution. Porosity was matched to this resolution using linear interpolation between measurements, except for the top and bottom depths, where we used the closest porosity data point. D_s_ was calculated from porosity based on Eq. 4, as the data quality of the measured formation factors was low. After matching the parameter depths, the CCSF-B scale (mbsf) was used for the modeling, which corrected CCSF-A for the core expansion during drilling by dividing the CCSF-A-depth by 1.06 (Expedition 323 Scientists, 2011). Cell count samples were retrieved from the Hole U1342B, published in Kallmeyer et al. (2012) and derived via cell extraction based on Kallmeyer et al. (2008) and SYBR Green I dye. If the samples were counted multiple times, we used the mean value. TOC data (weight %) were retrieved from the LIMS database and transformed into nmol C cm^−3^.

## 3. Results

### 3.1. Site descriptions

The Little Belt core (SKA14-05-B25) was co-located with IODP expedition 347 site M0059. Samples from the same site and day were designated SKA05-B25 by Deng et al. (2020), who describes the link between bioturbation and the microbial community. Information on benthic infauna and mineralization pathways can be found in Kristensen et al. (2018). The site was situated in a local depression prone to seasonal anoxia and, at the time of sampling, the bottom water had just barely re-oxygenated. Thus, the sediment was still thoroughly reduced and devoid of fauna (Kristensen et al., 2018). The sedimentation rate was 5 to 7 mm yr^−1^, based on the preliminary age-model of IODP Expedition 347 site M0059 retrieved from the same site (Andrén et al., 2015); paleoclimatic and paleoenvironmental data from the site are presented by Kotthoff et al. (2017). SKA14-05-B25 provided the youngest sulfate-reducing sediment available (average age ca 10 years), when sampling the upper 3.5 cm with cut-off syringes directly through the sediment–water interface in a box-core.

Greenland Glacier Fjord core SA13-ST8-47G was sampled in the innermost part of Nuup Kangerlua (Godthåbsfjord), close to the marine-terminating glaciers Narsap Sermia and Kangilinnguata Sermia [see Glombitza et al. (2015) and Pelikan et al. (2019) for details on all Greenland cores, including age models and geochemistry]. The glacier at the head of the fjord delivered large amounts of clastic material to the seabed, giving a mean sedimentation rate 2.9 cm yr^−1^ (95% confidence interval 2.2–4.1 cm yr^−1^ based on excess ^210^Pb activity) (Pelikan et al., 2019; Supplementary Figure S2), Thus, the 6-m long core provided samples with ages in the range of 0–200 years.

Greenland Main Fjord core (SA13-ST5-30G) was sampled in Nuup Kangerlua in the path of the ice-flow from the glaciers, but more distant from the glacier front, resulting in lower sedimentation rates and higher ages than the Glacier Fjord Core (SA13-ST8-47G). One bivalve shell was found at 39 cmbsf (cm below seafloor) and dated to 265 ± 42 cal yrs BP, a second at 553 cmbsf was dated to 457 ± 26 cal yrs BP. This dating could be ascribed to a dramatic change in sedimentation rate occurring sometime between the upper 39 cm (0.15 cm yr^−1^) and the rest of the core below (2.68 cm yr^−1^). A more likely explanation is that the shell found at 39 cmbsf was redeposited from an older deposit. Thus, the sediment accumulation rate was estimated to 1.2 cm yr^−1^ from linear extrapolation between the sediment surface and the age of the deepest shell (Supplementary Figure S2). According to this, the site provided samples in the age range from 80 to 600 years.

Greenland Side Fjord core (SA13-ST6-40G) was sampled in a side-fjord (Kapisillit Kangerluat) of Nuup Kangerlua; this side fjord is currently not connected to the Greenland ice-cap at its head. Matching of Rumohr cores and Gravity-cores indicated a loss of 10 cm from the top of the gravity-core. The core was dated to 4.6 ka cal. BP at 270 cmbsf, which implied an accumulation rate of 0.6 mm yr^−1^ (Supplementary Table S1). The surface sediment was heavily bioturbated and burrows were observed down to 43 cmbsf. The sediment was homogeneous in structure down 340 cmbsf, where the material changed from silty-clay to mud with an abundance of small ice-rafted pebbles. Data from below the deepest datable fossil at 270 cmbsf was rejected due to unknown age. The site provided usable sediment in the range from 200 to 4,600 years old (Supplementary Figure S2).

**Figure 1:**
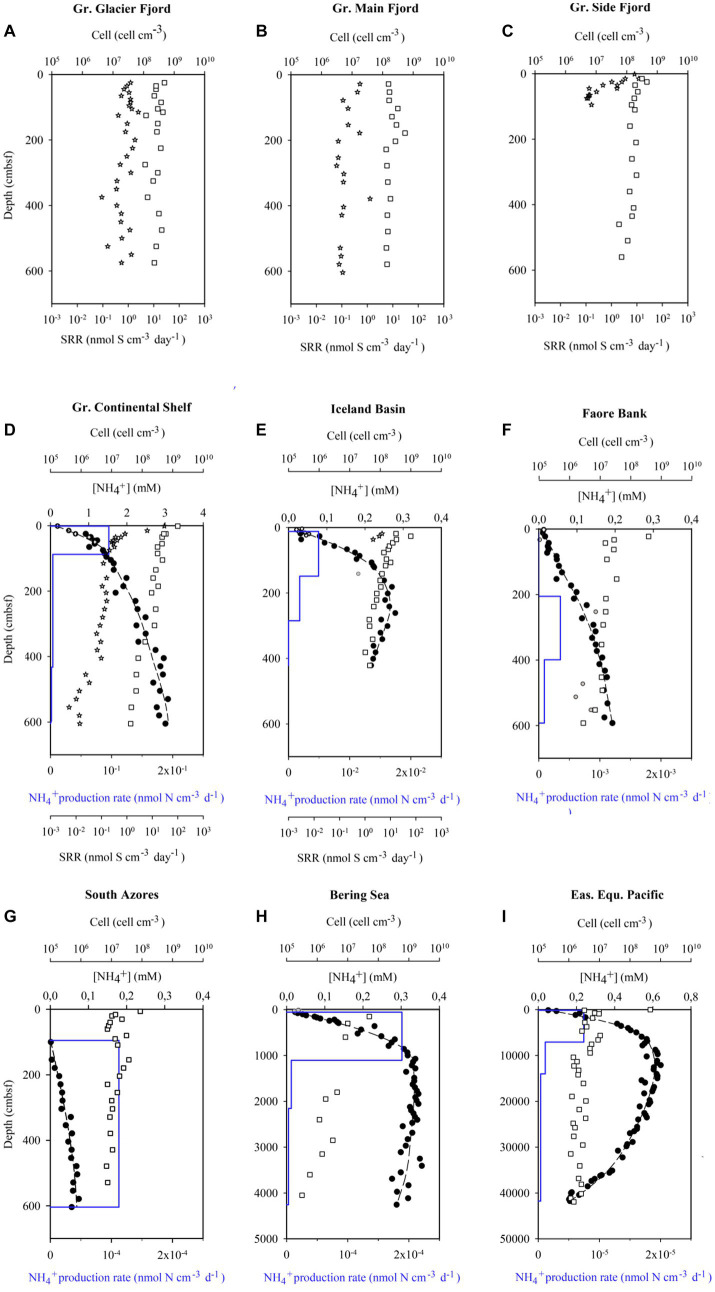
Nine of the datasets used to calculate mean cell-specific carbon oxidation rates showing pore water ammonium concentrations ([NH_4_^+^, filled circles]), model fits to the ammonium data (dashed line), modeled ammonium production rates (blue line), measured sulfate reductions rates (SRR, stars), and cell abundance (squares). Near-surface ammonium data marked with open circles was not included in the model fit. The South Azores model (G) starts at 0.95 mbsf because above this depth, ammonium concentrations were below the limit of quantification. Sulfate reduction rates from the Greenlandic cores were published in Glombitza et al. (2015), while the ammonium profiles were published in Pelikan et al. (2019). The data from Little Belt was not resolved in depth and, therefore, not plotted.

Iceland Basin gravity-core (DA12-11/1-GC01) and a box-core from the same site were collected from 2021 m deep water in the North Atlantic. The age model, the paleo-climate, and the paleo-circulation are described in Van Nieuwenhove et al. (2018), Orme et al. (2018), and Braun et al. (2017). Matching of pore-water profiles between gravity-core and box-core indicated a loss of 14 cm from the top of the gravity-core. The sediment was rich in carbonates and contained four distinct horizons of volcanic ash. Sulfate reduction rates were measured in the upper parts of the core with radiotracer, at sediment ages of 100–380 years. The sulfate reduction rates in the deeper parts of the core were below our level of quantification, but the ammonium profile in the entire core could be modelled (Figure 1E). Discarding the modelled rates of mineralization in the shallowest and deepest intervals left data with good confidence in the age-interval from 4,000 to 8,200 years (Supplementary Figure S2).

**Figure 2:**
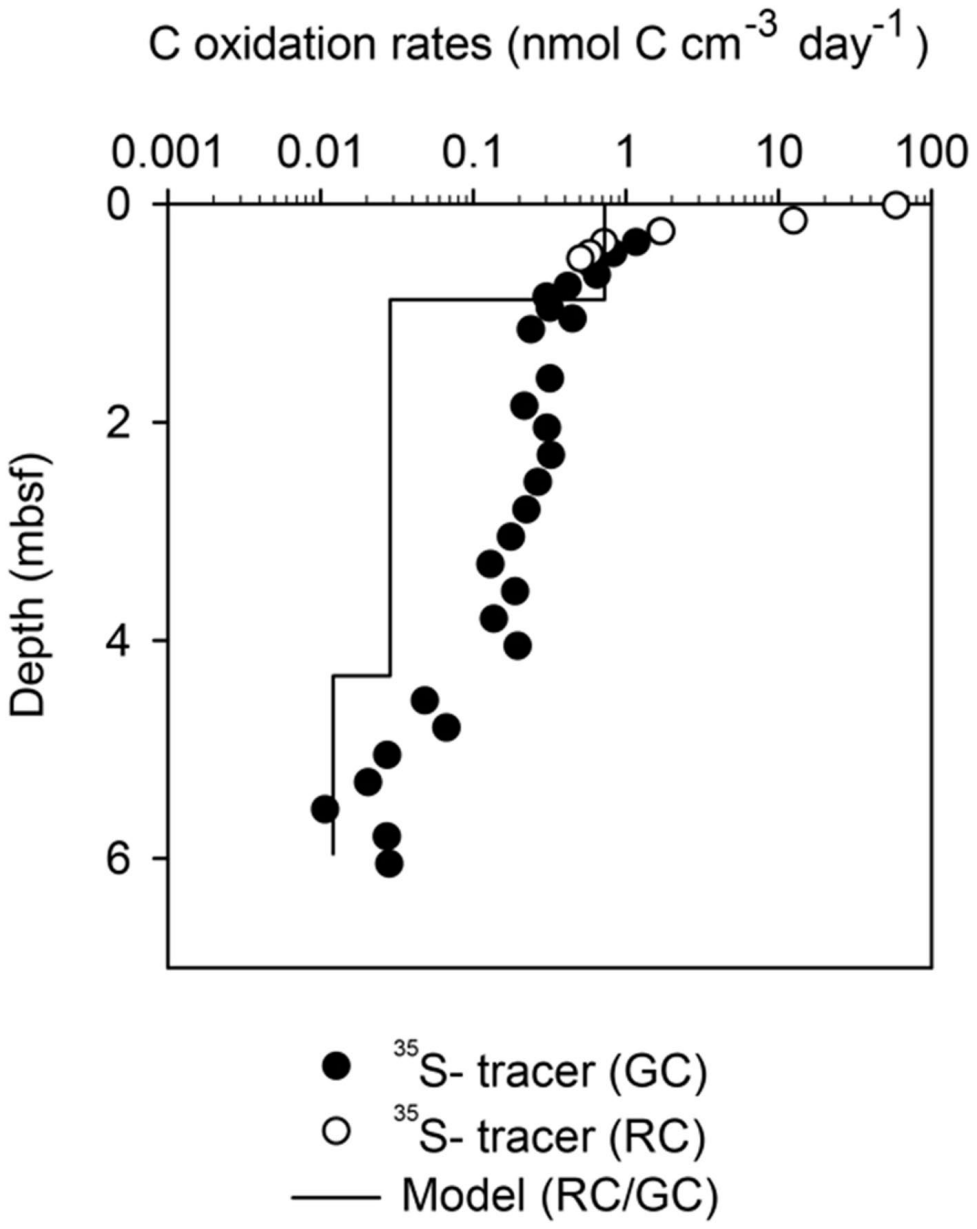
Comparison between carbon oxidation rates based on the modeling of ammonium concentration profiles, and carbon oxidation rates based on ^35^S-radiotracer incubation in Greenland Continental Shelf Sediments. The data was derived from Figure 1D by multiplying the sulfate reduction rates by 1.77 according to the C:S stoichiometry in Eq. 2 and multiplying the ammonium production rates by 7.59 according to the C:N stoichiometry from parameter plots (Supplementary Figure S4). RC is data from the Rumhor core and GC is data from the gravity-core.

Greenland Continental Shelf core (SA13-ST3-20G) and a Rumohr Lot core (SA13-ST3-17R) were both retrieved at the same site on the West Greenlandic Shelf in the Labrador Sea. Matching of the two cores indicated that the upper 18 cm was lost from the top of the gravity-core. The sediment accumulation rate was on average 0.48 mm yr^−1^, with a higher sedimentation rate during the recent 2,000 years (0.86 mm yr^−1^, 0–163.5 cmbsf, Supplementary Figure S2). From 253 to 568 cmbsf the core was weakly laminated, changing to millimeter-thick laminations (silty-sand) from 568 cmbsf to the bottom of the core, indicative of permanent ice cover during the Weichelian ice age (see Allan et al., 2021, for age model, stratigraphic and paleoenvironmental details). The core provided sulfate reduction rates at sediment ages ranging from 300 to 10,000 years and useful modeled rates based on pore water profiles of ammonium in the overlapping age range from 1,000 to 8,000 years old (Supplementary Figure S2; Figure 2).

Faroe Bank core (DA12-11-ST2-GC03) and a box-core were collected from 742 m deep water SW of the Faroe Islands. The sediments were carbonaceous with more than 14 cm of accumulated foraminiferal tests at the surface. Matching of Rumohr and gravity-cores indicated a loss of 18 cm from the top of the gravity-core. The sediment was too old for reliable determination of sulfate reduction rates with radiotracer but provided useful rates of carbon mineralization modeled from ammonium profiles in an age range from 22,000 to 35,000 years (Supplementary Figure S2, age model published in Braun et al., 2017).

South Azores core (DA14-ST1-GC01) was collected from 2,500 m deep water between the Azores Archipelago and the East Azores Fracture zone. The sediment consisted of calcareous deep-sea ooze covering the past 300,000 years. No attempts were made to measure reduction of ^35^S tracer as the younger layers of sediment, where tracer-incubations could have been feasible, were assumed not to be sulfate reducing. The model could not resolve more than one reaction zone from the ammonium profile. The calculated rate of mineralization was, therefore, only associated with the central third of the core where the age ranged from 100,000 to 200,0000 years (Supplementary Figures S2, S3).

Bering Sea IODP Leg 323 Bower Ridge Site U1342 was drilled in the southern Bering Sea under the Integrated Ocean Drilling Program (IODP). The data were retrieved from IODP LIMS Online Report http://web.iodp.tamu.edu/LORE/. The temperature at the seafloor was 1.9°C and increased by 0.0977°C m^−1^ down-core (Takahashi et al., 2011). The core covered the past 1,000,000 years, and mineralization of organic matter could be modelled with good confidence in the age-range from 400.000 to 700.000 years (Supplementary Figure S2).

Eastern Equatorial Pacific IODP Leg 201 Site 1,226 was drilled 300 km south of the Galapagos Islands. The temperature at the seafloor was 1.74°C and increased by 0.0572°C m^−1^ down-core (Shipboard Scientific Party, 2003; Wang et al., 2008). The core penetrated the 417 m thick sediment column all the way to the basaltic basement and covered the past 15,600,000 years. The core had an unusual geochemical profile due to diffusion of oxygen, nitrate, and sulfate from the basaltic basement (Jørgensen et al., 2006). Therefore, the core lacked a methanic zone and instead had a very deep penetration of sulfate. This unique core provided rates of carbon mineralization modelled from ammonium production in the sulfatic sediment in an age interval of 2–3.75 million years, after discarding data from the upper and lower reaction zones from PROFILE (Supplementary Figure S2).

### 3.2. Mineralization rates of organic carbon

**Figure 3:**
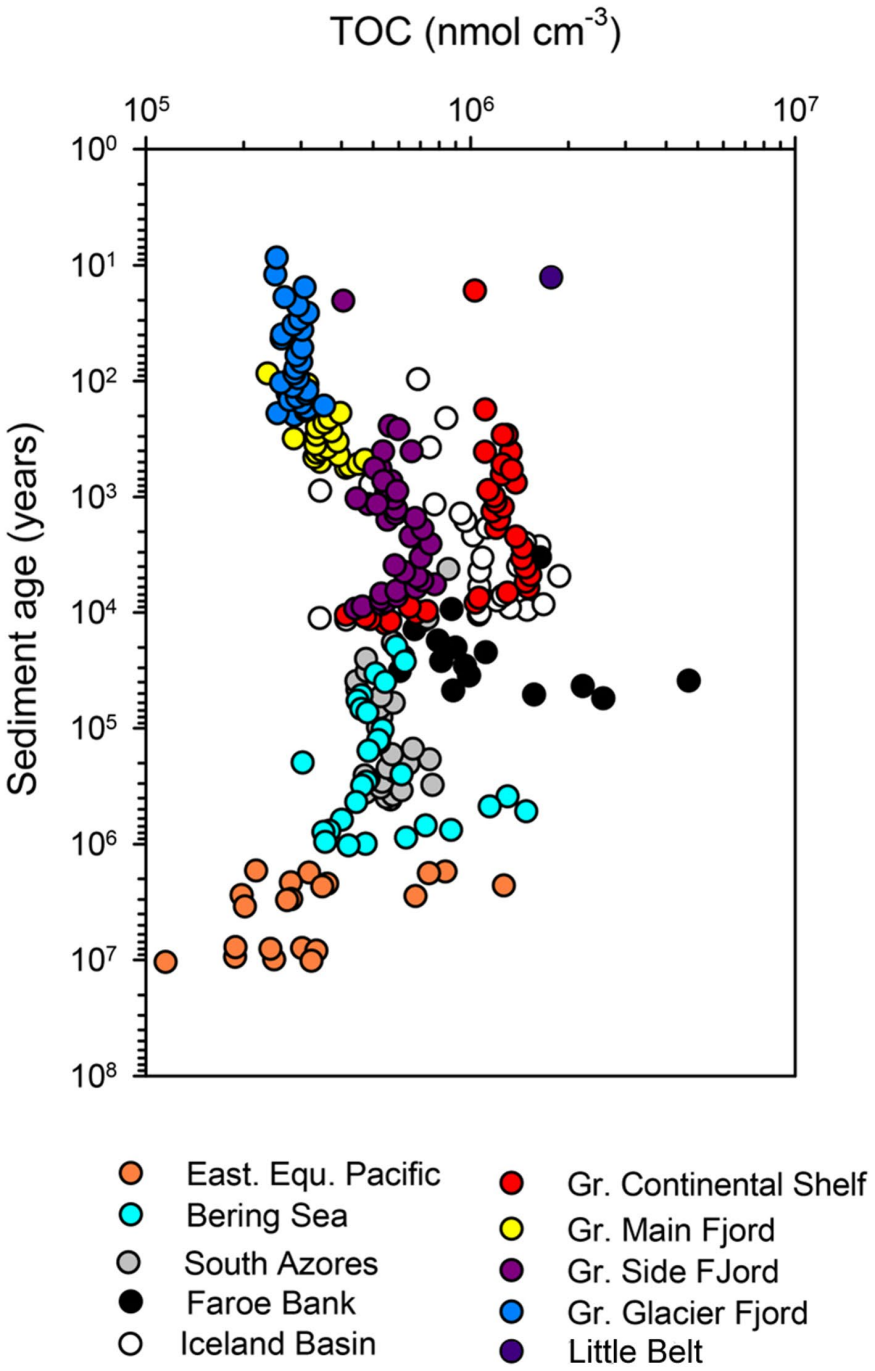
Total organic carbon concentration in all cores.

The content of organic carbon differed between sites but did not decrease systematically with increasing sediment age in the individual cores (Figure 3). The lowest carbon content was found near the glaciers in the Greenlandic fjord (site DA13-ST8-47G), where the organic matter was diluted by a large influx of glacially-derived clastic material, and in old deep-water deposits (ODP Leg 201/1226-B from the Eastern Equatorial Pacific). A continuous loss of organic material over time was partly balanced by compaction, when expressed in the volume-specific unit applied here, and the trajectory down-core was mostly determined by the depositional history. This is for example seen in the core from the Greenlandic continental shelf (SA13-ST3-20G), where the content of organic matter decreased abruptly in the early Holocene 6,000–10,000 years ago, when ocean circulation and climate changed dramatically after the Last Glaciation (the Weichselian; Figure 3). The total variation in organic matter content in the entire dataset was mostly between 2 × 10^5^ and 2 × 10^6^ nmol C cm^−3^.

**Figure 4:**
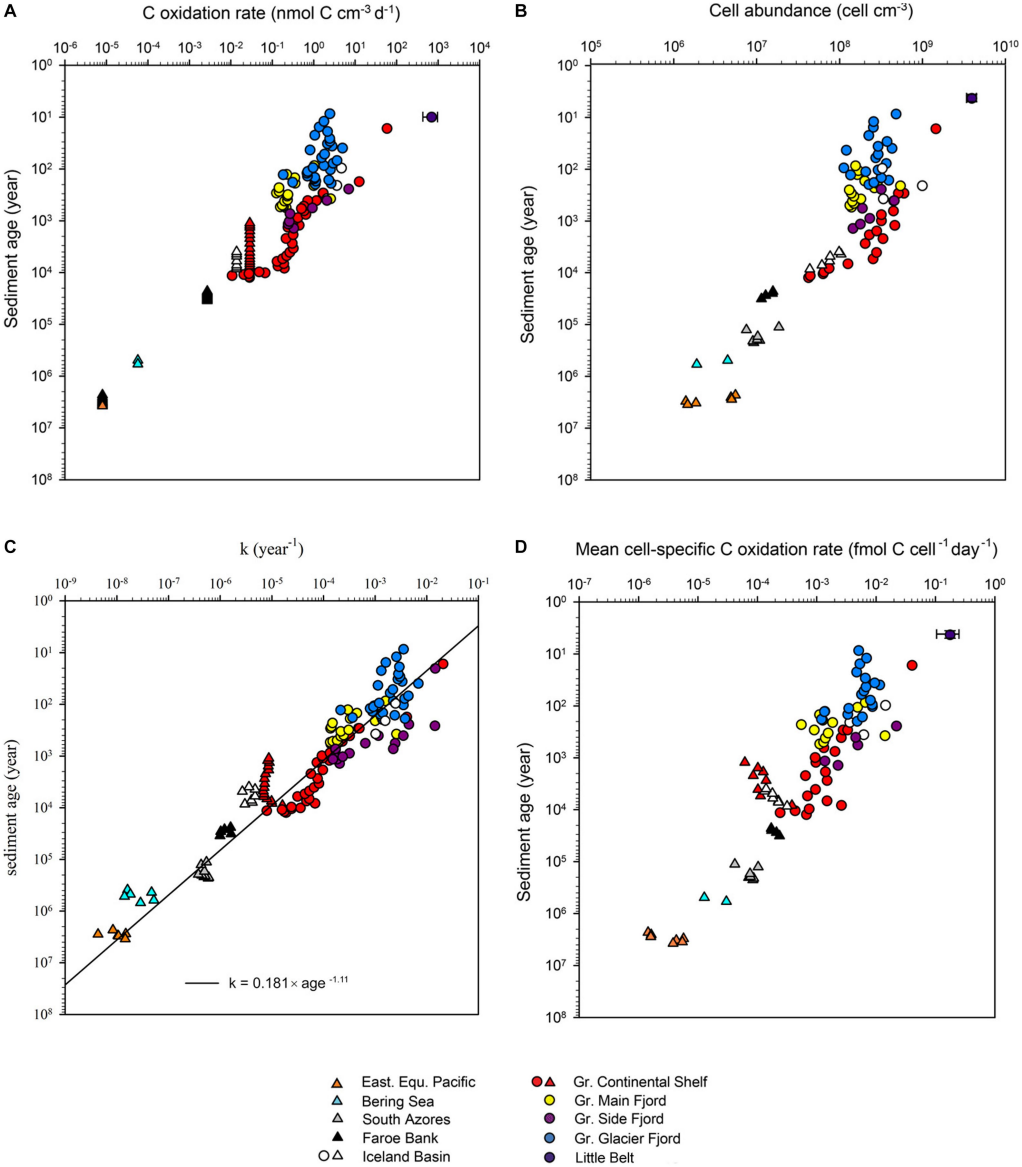
**(A)** Volume-specific organic carbon oxidation rates versus the age of the sediment. Datapoints represented by triangles were measured by radiotracer while datapoints represented by circles were measured by modeling of ammonium profiles. The error bar for Little Belt shows the standard deviation of multiple samples at the same depth. **(B)** Cell abundance versus age of the sediment. **(C)** Relation between the first order rate constant of organic matter mineralization (k) and the age of the sediment. The 95% confidence interval of the calculated exponent of the power function was −1.06 to −1.14 year^−1^. **(D)** Mean cell-specific carbon oxidation rates versus age of the sediment.

In sharp contrast to the low variability of organic carbon concentrations, the volume-specific carbon oxidation rates spanned 8 orders of magnitude from 7 × 10^2^ to 8 × 10^−6^ nmol C cm^−3^ day^−1^ (Figures 1A–I, 4A). The highest rates of mineralization of organic matter were found in the youngest sediments using ^35^S-radiotracer measurements. Conversely, the lowest rates of mineralization were determined in the oldest sediments using modeling of ammonium profiles. Fortunately, the geochemical settings on the Greenlandic shelf (SA13-ST3-20G) allowed both methods to be used on the same age interval (Figure 2). As expected, the modeled rates of ammonium production could not resolve the steep decrease in sulfate reduction in the upper meters, and the average rates given by the model systematically underestimated the measured rate of carbon mineralization in the top of the sediment and overestimated the rate in the lower half of the upper reaction zone. Deeper in the core, where the sulfate reduction rate changed more gradually, the two methods dropped in parallel, although with up to one order of magnitude discrepancy.

The reactivity of the organic matter, quantified as the momentary first order rate constant (k), decreased systematically with increasing age. There was no offset in the trend between data derived from incubations with radiotracer and data derived from modeling of ammonium profiles (Figure 4C). The best power-law fit to the relationship between k and sediment age was:


(13) $$k=0.180\times \mathrm{sediment}\;{\mathrm{age}}^{-1.11}\;$$

**Figure 5:**
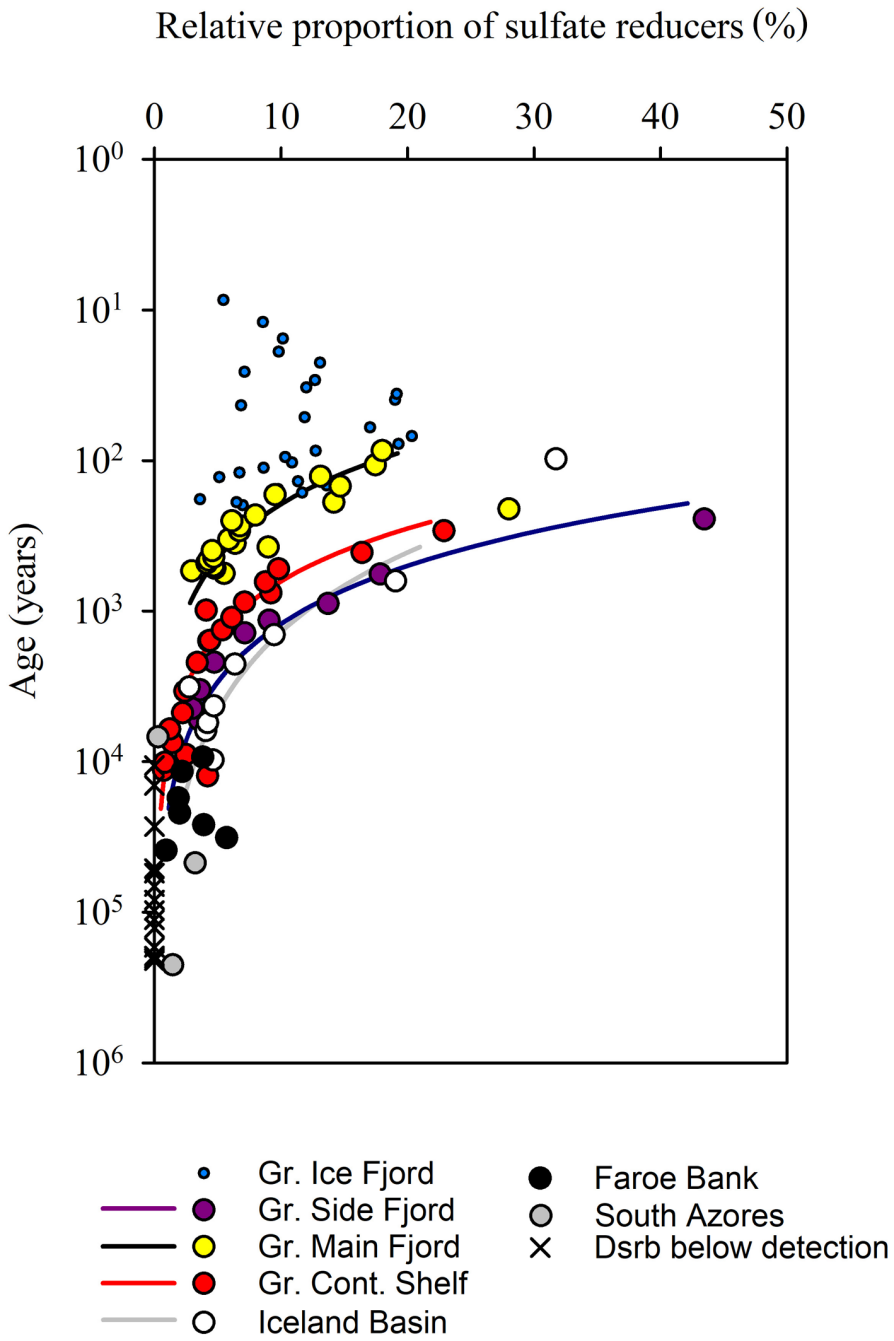
Relative proportion of sulfate-reducing microorganisms based on quantitative PCR of *dsrB* genes and *16S* rRNA genes from Bacteria and Archaea. In sediments older than 10^4^ years, sulfate reducers could not be detected in 14 out of 23 samples. The lines represent fits to power functions with exponents of −0.84 (−0.71 to 1.00) for Gr. Main Fjord, −0.86 for Gr. Cont. Shelf (−0.77 to −0.92), −0.78 (0.55 to 0.89) for Gr. Side Fjord and −0.57 (−0.36 to −72) for the Iceland Basin (95% confidence interval).

The exponent of −1.11 in the power function signified that the reactivity of organic carbon decreased by 1.11 order of magnitude each time the age increased by one order of magnitude. The exponent in the fit to the rate constant vs. depth (−1.11, Figure 5) was less negative than exponent in the fit to the sulfate reduction rate vs. depth (−1.23, Figure 4A). The faster drop in the rate of sulfate reduction compared to the rate constant was caused by a relatively fast initial drop in carbon concentration, and because loss of pore-space at depth concentrated the remaining volumetric carbon content.

### 3.3. Abundance of prokaryotic cells

The total cell abundance decreased with increasing sediment age, but only by three orders of magnitude, from 3.9 × 10^9^ to 1.9 × 10^6^ cells cm^−3^ (Figures 1A–I, 4B). The corresponding exponent of the fitted power function was −0.63. As the cell counts decreased relatively less than the volume-specific organic carbon oxidation rates (Figure 4A Figure 4B), the mean cell-specific carbon oxidation rate decreased continuously with sediment depth and age over ~5 orders of magnitude from 1.8 × 10^−1^ to 1.4 × 10^−6^ fmol C cell^−1^ day^−1^, with no indication that the decrease was tapering off in the oldest sediment (Figure 4D).

The relative proportion of *dsrB* vs. *16S* rRNA gene copies, and thus the proportion of potentially sulfate-reducing microorganisms within the total microbial community, was up to 40% in the youngest sediments but decreased rapidly with increasing sediment age (Figure 5). In sediments older than 10,000–100,000 years, the abundance of *dsrB* gene copies fell below the limit of quantification (10^4^ cm^−3^). The decreasing proportion of sulfate-reducing microorganisms did not follow a common function with increasing age, but if the individual cores were treated separately, then the decrease followed power functions with exponents of −0.77 to −0.86 (Figure 5). We disregard the calculated exponent from the Iceland Basin here because the surface of this deep-sea core was most likely iron-reducing rather than sulfate reducing, and because the high calculated percentages of sulfate-reducing cells deep in the sediment were interspersed with samples below the level of quantification which skewed the fit.

The cell numbers decreased with an exponent of −0.63 and the proportion of sulfate-reducing cells decreased with an exponent of −0.82. Combined, this caused the number of sulfate-reducing cells to decrease with an exponent of −1.45, which is closely matched by the rate at which the mineralization of organic carbon decreased (exponent of −1.36). Thus, the combined effect of decreasing cell numbers and decreasing proportion of sulfate-reducing cells caused the quantified abundance of sulfate-reducing cells to decrease as steeply as the rate of carbon mineralization over sediment ages from 10^1^ and at least to 10^4^ years old. Accordingly, the sulfate reduction rates per sulfate-reducing cell remained surprisingly constant in the order of 10^−2^ fmol sulfate cell^−1^ day^−1^, although the overall cell-specific community metabolism decreased by 4 orders of magnitude. We could not resolve how the sulfate reduction rate per sulfate-reducing cell developed in even older sediments ranging from 10^4^ up to 10^7^ years because our methods were not sensitive enough to quantify the number of *dsrB* gene copies.

## 4. Discussion

### 4.1. Reaction rate and reactivity of organic matter in old sediments

The careful selection of sites and methods allowed us to determine the rate of mineralization of organic matter in sulfatic sediments (Canfield and Thamdrup, 2009; Jørgensen, 2021) with ages from 10 years to 3,750,000 years. The data extended the power law of organic matter decay constants, first presented by Middelburg (1989), to even older sediments and overcame the original need for a site-specific “initial age,” possibly because our data were more uniform with respect to temperature and geochemical zone. In contrast to previous data syntheses [e.g., Middelburg (1989) and Katsev and Crowe (2015)], our approach did not rely on a measurable decrease in the concentration of organic matter with increasing depth and age of the sediment. In fact, we found little correlation between depth in the sediment column and the concentration of organic matter (Figure 3). Our correlation predicted a slightly faster loss of reactivity with k = 0.180 × sediment age^-1.11^ (Eq. 13), compared to models based on, or verified from, vertical profiles of organic carbon, which have exponents ranging from −0.8 to −1 (Shang, 2023). Power-law models with exponents close to −1 demonstrate that the overwhelming factor responsible for decreasing rates of microbial catabolism in ageing sediments is not so much the loss of organic carbon, but rather the loss of organic carbon reactivity (Figures 3, 4D). Conceptually, this corresponds well with the increasing molecular complexity of degrading organic matter (e.g., Estes et al., 2019; Hach et al., 2020), but poorly with models that assume concurrent degradation of multiple pools of organic matter with individual degradability (Jørgensen, 1978b; Shang, 2023). Note, however, that the two types of models provide equally good fits to empirical data (Arndt et al., 2013).

### 4.2. The link between sediment age, community size, and respiration rate

Cell numbers decreased as a function of sediment depth and age, as seen in several previous studies (Whitman et al., 1998; Kallmeyer et al., 2012). The decrease in total cell abundance with increasing age of the sediment was much slower than the decrease in carbon oxidation rates (Figure 4A vs. Figure 4B), which implied a large drop in the mean cell-specific rates of catabolism (Figure 4D). The degradation of organic matter in sulfatic marine sediments is, however, divided between two major guilds of cells, one guild that hydrolyses and ferments complex organic matter to volatile fatty acids and hydrogen, and a second guild that oxidizes these fermentation products to CO_2_ and water while reducing sulfate to sulfide. The rate-limiting step in mineralization of complex organic matter is the initial hydrolysis (Beulig et al., 2018) and the fermenters will take up the resulting monomers so efficiently that their concentrations are mostly too low to even detect. The community that oxidizes the fermentation products via anaerobic respiration is equally efficient, and fermentation products do not normally accumulate above a few μM (Postma and Jakobsen, 1996; Wang et al., 2010; Glombitza et al., 2015). The metabolic guild that has the most energetic respiratory metabolism will deplete the fermentation products to such low concentrations that energy conservation via proton translocation across the cell membrane is only barely possible. This excludes energy conservation from respiration with less energetic electron acceptors and causes the characteristic geochemical redox-zonation with limited overlap between utilization of different external electron acceptors (Postma and Jakobsen, 1996). We can, therefore, assume a tight link between fermentation and sulfate reduction regardless of which specific fermentation processes that were active: The fermenters oxidize some organic carbon to CO_2_, while concurrently producing more reduced carbon or H_2_ in the process. When these electron-rich substrates are then used by the sulfate reducers, it balances the stoichiometric ratio between sulfate consumption and total CO_2_ production to the same value as if the sulfate reducer had mineralized the original organic matter with no fermentation involved (Eq. 2). This way, the electrons from complex organic matter must pass through both fermentation and sulfate reduction for the carbon to be funneled fully to CO_2_, and the rate of sulfate reduction is a measure of the flow of carbon and electrons through both processes. Even syntrophic interactions with direct electron transfer do not change the overall stoichiometry between mineralized organic carbon/CO_2_ and sulfate.

The fermentation product acetate is instrumental in the transfer of reducing equivalents from fermentation to sulfate reduction, and oxidation of acetate accounts for 30–65% of the sulfate reduction rate in sulfatic sediments (Christensen and Blackburn, 1982; Finke et al., 2007; Beulig et al., 2018). We specifically selected sediments for the study where all other electron accepters than sulfate and CO_2_ had already been depleted. Under these conditions, sulfate reduction is the primary terminal electron accepting process, and all known prokaryotes that respire via sulfate use the *dsrB* gene. The gene is also used in sulfide oxidation, but the sediment strata that we analyzed did not contain suitable electron accepters for this process. We therefore assumed that all cells that contained the *dsrB* gene were potential sulfate reducers, and as they were found in sulfate-reducing sediment, we assumed that all potential sulfate reducers were active. This allowed us to calculate the per-cell sulfate reduction rate. We found elevated rates of per-cell sulfate reduction in upper centimeters of sediment with active bioturbation. But when both the decreasing cell numbers and the decreasing proportion of sulfate reducers were considered, the cell-specific carbon oxidation rate by sulfate-reducing microorganisms in the subsurface stayed remarkably constant on the order of 10^−2^ fmol C cell^−1^ day^−1^, despite increasing depth and a drop in sulfate reduction rates of four orders of magnitude (Figure 5).

Previous studies have shown relatively high cell-specific rates of sulfate reduction in the very surface of coastal sediments, gradually decreasing to an organic carbon oxidation rate of 10^−3^ to 10^−2^ fmol C cell^−1^ day^−1^ between 30 and 100 cm below seafloor (Hoehler and Jørgensen, 2013; Petro et al., 2019). Interpretation of the data deeper in the sediment from these coastal sites is difficult because of the transition from the sulfatic zone and into the methanic zone, and because the early qPCR protocols and primers used (Leloup et al., 2007, 2009) lacked the necessary resolution. In an attempt to predict the cell specific rates of sulfate reduction deeper in the sediment, Lever et al. (2015) extended the estimate of Hoehler and Jørgensen (2013) into deeply buried sediments of the Peru Margin by assuming that sulfate reducers constituted 10% of the total microbial community regardless of sediment depth and age. This assumption resulted in calculation of constantly decreasing per-cell rates of sulfate reduction in deeper sediment, but later studies have not confirmed the proportion of sulfate reducers to be constant (Webster et al., 2006; Petro et al., 2019). Thus, the constant cell specific sulfate reduction rates across depth and age up to 20,000 years seen in our study do not contradict the more recent studies and might imply that the size of the sulfate-reducing community is indeed controlled by a fixed minimum energy requirement of these microorganisms, as hypothesized by Hoehler and Jørgensen, (2013). Further, this minimum metabolic rate of sulfate reducers in deeply buried marine sediments might not be far from that seen below the depth of bioturbation in sediment that is buried only 30 cm deep and is only 300 years old (Petro et al., 2019).

Our attempt to detect sulfate-reducing prokaryotes in sediments older than 26,000 years was not successful as the *dsrB* gene copy numbers fell below our limit of quantification of ~10^4^ gene copies cm^−3^. Thus, it awaits more sensitive experimental methods to see if the proportion of sulfate reducers continue to drop with increasing sediment age beyond 26,000 years, which is necessary for the cell specific sulfate reduction rates to stay at the constant level of 10^−2^ fmol C cell^−1^ day^−1^ that we observed down to this depth. There are, however, indications that this could indeed be the case: Studies have revealed (a) low abundance of functional genes related to sulfate reduction (*dsrAB* genes) or methanogenesis (methyl-coenzyme M reductase, *mcr* genes) in deep sediments (<1% of total community, Schippers and Neretin, 2006; Lever, 2013), (b) fermentation-related genes that were much more abundant than *dsr* or *mcr* genes in metagenomes (Kirchman et al., 2014; Gaboyer et al., 2015), and (c) apparent virtual absence of genes related to sulfate reduction in metagenomes from deep sulfatic sediments of the Bering Sea (Biddle et al., 2008). The apparent difficulty in detection and quantification of dsr genes in the diverse pool of DNA extracted form sediment from deep below the sea floor on the Peru Margin indicate that terminal-oxidizing prokaryotes are present at low abundance (Webster et al., 2006), while the consistent detection of mRNA transcript of dsr (Orsi et al., 2013, 2016) indicate that sulfate reduction play a larger role in community activity than the low proportion of sulfate-reducing prokaryotes suggests.

### 4.3. Division of Gibbs free energy between fermentation and sulfate reduction

To assess the division of Gibbs free energy (ΔG_r_) between fermenters and sulfate reducers, we calculated the Gibbs free energy (ΔG_r_) by complete oxidation of organic matter to CO_2_ via Eqs 11, 12 according to LaRowe and Van Cappellen (2011). We also calculated ΔG_r_ for oxidation of acetate to CO_2_ with sulfate as electron acceptor based on measured concentrations of reactants and products (Eqs 12, 13). By subtracting the energy yield of this terminal oxidation from the total energy yield, we could estimate the energy yield of fermentation under *in situ* conditions without needing to know the molecular identity or the concentrations of fermentation substrates (i.e., the products of hydrolysis). This is a rather crude approximation, as fermentation processes produce other products than acetate, and sulfate reducers oxidize H_2_ and other volatile fatty acids, such as formate or propionate, in addition to acetate (Glombitza et al., 2015). Moreover, this calculation overestimates the energy available for the fermenters, as the free energy from extracellular hydrolysis cannot be coupled to energy conservation (i.e., to ATP). Note also that our operational definition of “fermenters” includes all organisms involved in production of acetate regardless of the actual biochemical pathway. Thus, the purpose was not to calculate the accurate ΔG_r_ of the processes or to compare energy yields close to thermodynamic thresholds. But to test if the overwhelming dominance of fermenting cells could be explained by an inequal sharing of energy between fermentation and sulfate reduction. Thus, we calculated the division of energy between fermentation and sulfate reduction on the four Greenland cores and in the oldest core from the Eastern Equatorial Pacific, where we had the most complete data on pore water chemistry that is needed.

In the core from the Greenland Continental Shelf (SA13-ST3-20G), the ΔG_r_ liberated by fermentation 1 m below seafloor was −43 kJ (mol C)^−1^, while the ΔG_r_ liberated by acetate oxidation was −24 kJ (mol C)^−1^. At 5.9 meters below seafloor, the values changed only slightly to −42 kJ (mol C)^−1^ and − 18 kJ (mol C)^−1^, respectively. The remaining Greenlandic cores (SA13-ST5-30G, SA13-ST6-40G, SA13-ST8-47G) were in the same range, with even less variation down-core. In the core from the Eastern Equatorial Pacific (ODP Leg 201, Hole 1,226-B), the fermenters and the terminal oxidizers shared the energy in a similar manner with −42 to −43 kJ (mol C)^−1^ for the fermenters and − 35 to −26 kJ (mol C)^−1^ for the sulfate reducers. Similar values of −42.9 ± 2.7 kJ mol acetate^−1^ have previously been reported for sulfate reduction coupled to acetate oxidation for most of the sediment column at ODP Site 1226 (Wang et al., 2010), and − 32 kJ mol C^−1^ for global marine sediments in general (Bradley et al., 2020).

The assumptions and approximations in the thermodynamic calculations above were coarse. Most notably the assumed temperature of 25°C, while the effects of the pressure between one bar and *in situ* were negligible (Helgeson, 1969). But effects of pressure and temperature on thermodynamic calculations are much less severe than the effects on kinetics (Jannasch, 1997), and even the temperature effect does not influence the conclusions drawn here: The standard Gibbs energy of sulfate reduction per mole acetate, for example, only change from −48.1 to −44.5 kJ (mol acetate)^−1^ between reference temperature and pressure (25°C, 1 bar) and *in situ* conditions (2°C, 50 bar) at the Greenlandic sites. Thus, the coarse calculations still allow us to conclude that ΔG_r_ from mineralization of organic carbon to CO_2_ was shared between the guilds of fermenters and terminal oxidizers in a ratio of roughly 1:1 regardless of site and sediment age. Since the fermentation products do not accumulate in the sediment, there must be a balance between fermentation and sulfate reduction, whereby rates of fermentation limit and control the rates of sulfate reduction (Jørgensen, 2021). Thus, the carbon flow through fermentation must be similar to the carbon flow through sulfate reduction. As non-sulfate-reducing bacteria outnumbered the sulfate reducers by >100-fold, the energy dissipation (i.e., power) available to the individual fermenters was much lower than the power available to the individual sulfate reducers.

Volume-specific power of reactions in the sediment (Watt cm^−3^ or Joule s^−1^ cm^−3^) was calculated by multiplying ΔG_r_ of the reaction (J mol^−1^\C) by the rates of reaction (mol C cm^−3^ s^−1^). The mean cell-specific sulfate reduction rates in the Greenland cores were in the order of 10^−2^ fmol C cell^−1^ day^−1^, which was not far outside the range from 10^−1^ to 10^1^ fmol C cell^−1^ day^−1^ seen in cultures of mesophilic and psychrophilic sulfate-reducing bacteria (Canfield et al., 2000). The 10^−2^ fmol C cell^−1^ day^−1^ translates to a power dissipation of 10^−17^ W cell^−1^ if we assume an energetic yield of −42 kJ (mol C)^−1^ (see above and note that J/s equals W). This rate of energy dissipation is similar to the value calculated for coastal surface sediments by Lever et al. (2015), and similar to the per-cell power dissipation in slow-growing axenic cultures of *Desulfotomaculum putei* (Davidson et al., 2009). It is also similar to the power dissipation of extremely low-light adapted green sulfur bacteria in the Black Sea (1.9 × 10^−17^ W cell^−1^; Marschall et al., 2010). Conversely, the values are far above the per-cell power dissipation calculated for deeply buried sulfate reducers on the Peru Margin by Lever et al. (2015), even though our estimation of the carbon oxidation rates in the same deep Pacific sediment based on NH_4_^+^ agrees well with calculations based on sulfate (Wang et al., 2008). The difference between our relatively high and invariant estimate of power dissipation by deeply buried sulfate reducers, and the low and age-dependent power dissipation reported by Lever et al. (2015) is that these authors assumed a constant 10% proportion of sulfate-reducing microorganisms (see above). The fact that the power dissipation we calculated per sulfate-reducing cell did not continue to drop with depth and age in the sediment, is because the estimated number of sulfate reducers decreased in near perfect balance with the decrease in sulfate reduction rates. This could indicate that 10^−17^ W cell^−1^ approaches a minimum power requirement of sulfate reducers in deeply buried sediments. As the same range of power dissipation can be observed only 30 cm below the sediment–water interface in eutrophic coastal sediments and in axenic cultures, this suggests that the sulfate reducers in the deep biosphere do not necessarily have any unique physiological adaptations to low energy availability.

The cell-specific power dissipation of extremely old oxic sediments has been calculated to 5 × 10^−18^ W cell^−1^ in the North Pacific Gyre, and within the range from 3.5 × 10^−18^ down to 4.9 × 10^−20^ in the South Pacific Gyre (LaRowe and Amend, 2015a). These calculations assume an energetic yield of −443 kJ (mol C)^−1^ for aerobic oxidation of organic matter all the way to CO_2_ in a single step (Lever et al., 2015). The low values from the SPG were calculated based on an assumed constant rate of carbon burial across the 75 million years and is, most likely, less accurate than the data from the North Pacific Gyre (NPG) that was based directly on modelled oxygen consumption rates (Røy et al., 2012). Thus, the apparent maintenance power of aerobic and anaerobic respiration come within one order of magnitude of each other. And in contrast to the per cell power dissipation of the “fermenters” (or the total carbon oxidation per total cells), both aerobic and anaerobic respiration appears to converge at fixed rates of per cell power dissipation rather than dropping continuously with increasing sediment age. The difference in the two levels is not understood, as the higher energetic cost of biomolecule synthesis aerobes (McCollom and Amend, 2005) would suggest that the minimum maintenance power of aerobes should be larger than that of anaerobes.

It is possible to estimate the power dissipation by the guild of fermenters in the deep sulfatic sediments older than 26,000 years that we studied, even though the proportion of fermenters vs. terminal oxidizers was not known. This is because the number of fermenters is essentially equal to the total cell numbers, only offset by 1% sulfate reducers or less. In contrast to the situation for sulfate reducers, the per cell power dissipation for the fermenters did not reach a plateau that could indicate a minimum power requirement of fermenting cells, although such a limit must exist. The lowest values reached in our dataset was 7 × 10^−22^ W cell^−1^. This rate of power dissipation is so low that the cells would just barely cover the lowest estimate of the repair cost associated with spontaneous racemization of aspartic acid (Lever et al., 2015). But there are no indications in the data that 10^−6^ fmol C cell^−1^ d^−1^, corresponding to 10^−22^ W cell^−1^ is the lower limit, it is simply where our dataset ends. In principle, the correlation could extend further back in time. But extrapolation of log–log plots back in time from 4 million years would rapidly reach the age of Planet Earth.

In conclusion, we found that the population size of sulfate-reducing microorganisms was in balance with the rate of sulfate reduction in sub-surface sediments. The minimum per-cell sulfate reduction rate, and thus the minimum per cell power dissipation, in 10,000 to 100,000 years old sediment was not radically different from the much shallower sub-surface. Thus, we do not expect sulfate reducers in the deep subsurface to have a fundamentally different maintenance metabolism compared to organisms thriving less than 1 m below the seafloor in coastal seas (Starnawski et al., 2017). We did not see a lower threshold for metabolic activity of cells not involved in terminal oxidation, although there was a clear log–log correlation between the total number of cells and the rate of carbon mineralization. It is unknown which physiological properties make the fermenters able to apparently subside with a minute fraction of the power needed for sulfate reducers. The large number of cells relative to the extremely low rates of mineralization in the deep biosphere therefore remain enigmatic.

## Data availability statement

The original contributions presented in the study are included in the article/Supplementary material, further inquiries can be directed to the corresponding author.

## Author contributions

HR and BJ designed the study. HR, CP, M-SS, BL, KK, and MJ retrieved the cores and collected pore water and the solid phase samples. MJ performed geochemical, microbiological analyses, and thermodynamic estimations. KK supervised the molecular work. M-SS, and CP built the age models. MJ and HR wrote the manuscript. All authors contributed with comments, corrections, and discussions.

## Funding

This work was supported by the Graduate School of Science and Technology at Aarhus University, the Danish National Research Foundation [n° DNRF104], the European Research Council, EU FP7 ERC Advanced Grant (n° 294200, MICROENERGY). Cruise funding was provided by the Danish Center for Marine Research and the Arctic Research Centre of Aarhus University. M-SS was supported through the European Union’s Horizon 2020 research and innovation program under grant agreement no. 869383 (ECOTIP) (M-SS) and the Independent Research Fund Denmark (grant no. 0135-00165B GreenShelf).

## Conflict of interest

The authors declare that the research was conducted in the absence of any commercial or financial relationships that could be construed as a potential conflict of interest.

## Publisher’s note

All claims expressed in this article are solely those of the authors and do not necessarily represent those of their affiliated organizations, or those of the publisher, the editors and the reviewers. Any product that may be evaluated in this article, or claim that may be made by its manufacturer, is not guaranteed or endorsed by the publisher.

## Footnotes

^1^ http://www-odp.tamu.edu/database/
