# Supplementary material for: Cell-specific rates of sulfate reduction and fermentation in the sub-seafloor biosphere
Source: Front Microbiol. 2023 Jul 24;14:1198664. doi: 10.3389/fmicb.2023.1198664 (PMC10405931; doi:10.3389/fmicb.2023.1198664)
Supplement: Supplementary file 1 [file Data_Sheet_1.docx]

**PRIMERS AND QPCR DETAILS**

The dsrB gene qPCR assay was based on the primer variant mixtures dsrB-F1a-h and dsrB-RSI1a-f (Lever et al. 2013) (all 5´-3´) : dsrB F1a: CAC ACC CAG GGC TGG, dsrB F1b: CAT ACT CAG GGC TGG, dsrB F1c: CAT ACC CAG GGC TGG, dsrB F1b: CAC ACT CAA GGT TGG, dsrB F1e: CAC ACA CAG GGA TGG, dsrB F1f: CAC ACG CAG GGA TGG, dsrB F1g: CAC ACG CAG GGG TGG, dsrB F1h: CAT ACG CAA GGT TGG, dsrB 4RSI1a: CAG TTA CCG CAG TAC AT, dsrB 4RSI1b: CAG TTA CCG CAG AAC AT, dsrB 4RSI1c: CAG TTG CCG CAG TAC AT, dsrB 4RSI1d: CAG TTT CCG CAG TAC AT, dsrB 4RSI1e: CAG TTG CCG CAG AAC AT, dsrB 4RSI1f: CAG TTT CCA CAG AAC AT.

The bacterial 16S rRNA gene qPCR assay was based on the primer pair Bac908F 5´-AAC TCA AAK GAA TTG ACG GG-3´ (modified from Ohkuma & Kudo 1998) and Bac1075R 5´-CAC GAG CTG ACG ACA RCC-3´ (Ohkuma & Kudo 1998). The archaeal 16S rRNA gene qPCR assay was based on the primer pair Arch915Fmod 5´-AAT TGG CGG GGG AGC AC-3´ (Cadillo-Quiroz et al. 2006) and Arch1059R 5´-GCC ATG CAC CWC CTC T-´3 (Yu et al. 2005).

**REFERENCES**

Cadillo-Quiroz H, Bräuer S, Yashiro E, Sun C, Yavitt J, Zinder S. 2006. Vertical profiles of methanogenesis and methanogens in two contrasting acidic peatlands in central New York State, USA. Environ Microbiol. 8:1428-1440

Lever MA, Rouxel O, Alt JC, Shimizu N, Ono S, Coggon RM, Shanks WC, Lapham L, Elvert M, Prieto-Mollar X, Hinrichs K-U, Inagaki F, Teske A. 2013. Evidence for microbial carbon and sulfur cycling in deeply buried ridge flank basalt. Science 339:1305–1308. https://doi.org/10.1126/ science.1229240.

Ohkuma M, Kudo T. 1998. Phylogenetic analysis of the symbiotic intestinal microflora of the termite Cryptotermes domesticus FEMS Microbiology Letters 164: 389–395.

Yu Y, Lee C, Kim J, Hwang S. 2005. Group-specific primer and probe sets to detect methanogenic communities using quantitative real-time polymerase chain reaction. Biotechnol Bioeng. 89:670-679.


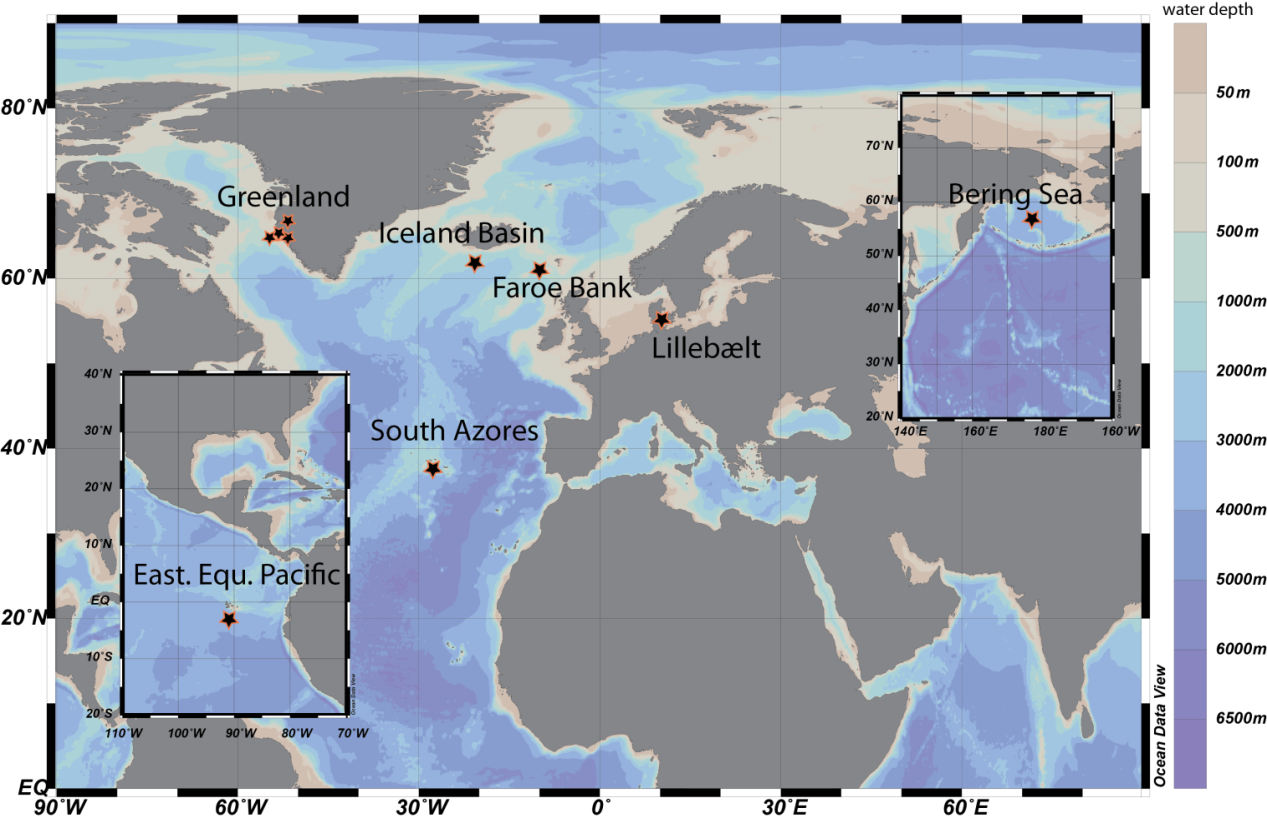
**Suppl. Figure S1:** Sampling sites plotted with the free software Ocean Data View (https://odv.awi.de/). Sites can also be seen directly on Google map https://drive.google.com/open?id=1rYE3drQ6eSkWIjRFQtpxwL265bM&usp=sharing


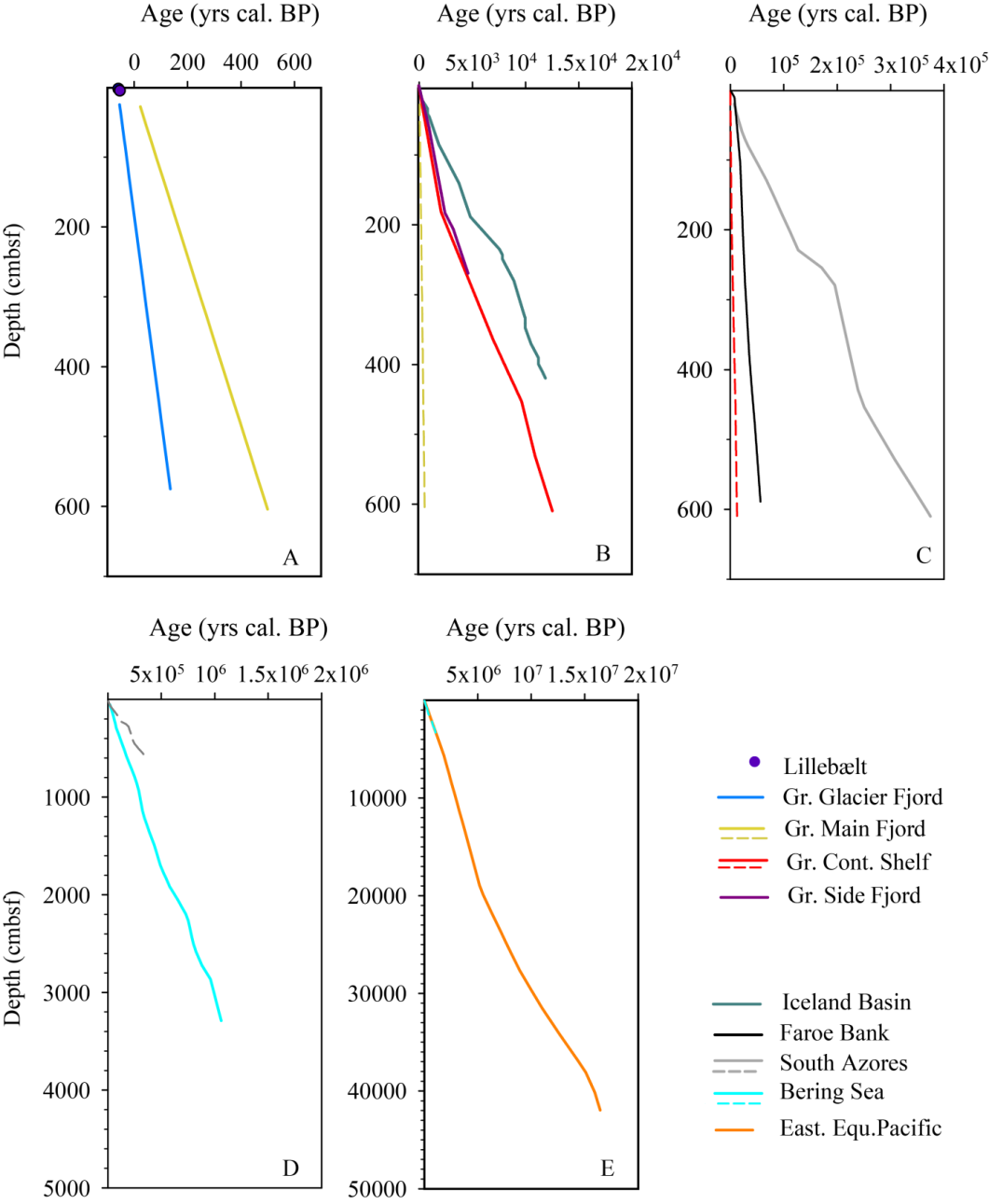
**Suppl. Figure S2:** Relationship between age before present (BP) and depth of the studied sites. From left to right (A to E), the youngest sediment cores to the oldest. Note the difference in the age and depth scale between the different plots, which are visible with dashed lines reported from the precedent plot (D and E). Note also that the age based on sedimentation rates (Little Belt and Gr. Glacier Fjord) were recalculated into years before present (BP).

**Suppl. Figure S3:** Age model of the South Azores core. A) Ca/Fe ratios derived from XRF core scanning matched to the global δ^18^O stack of benthic foraminifera isotope LR04 (Liesicki and Raymo, 2005, A Pliocene-Pleistocene stack of 57 globally distributed benthic 18O records, Paleoceanography, 20, PA1003, doi:10.1029/2004PA001071)). The Marine Isotope Stages (MIS’s) are indicated on the plot based on the corresponding δ^18^O peaks. The odd numbers are associated to warmer interglacial periods. B) Depth-age model based on the ages of the MIS’s.


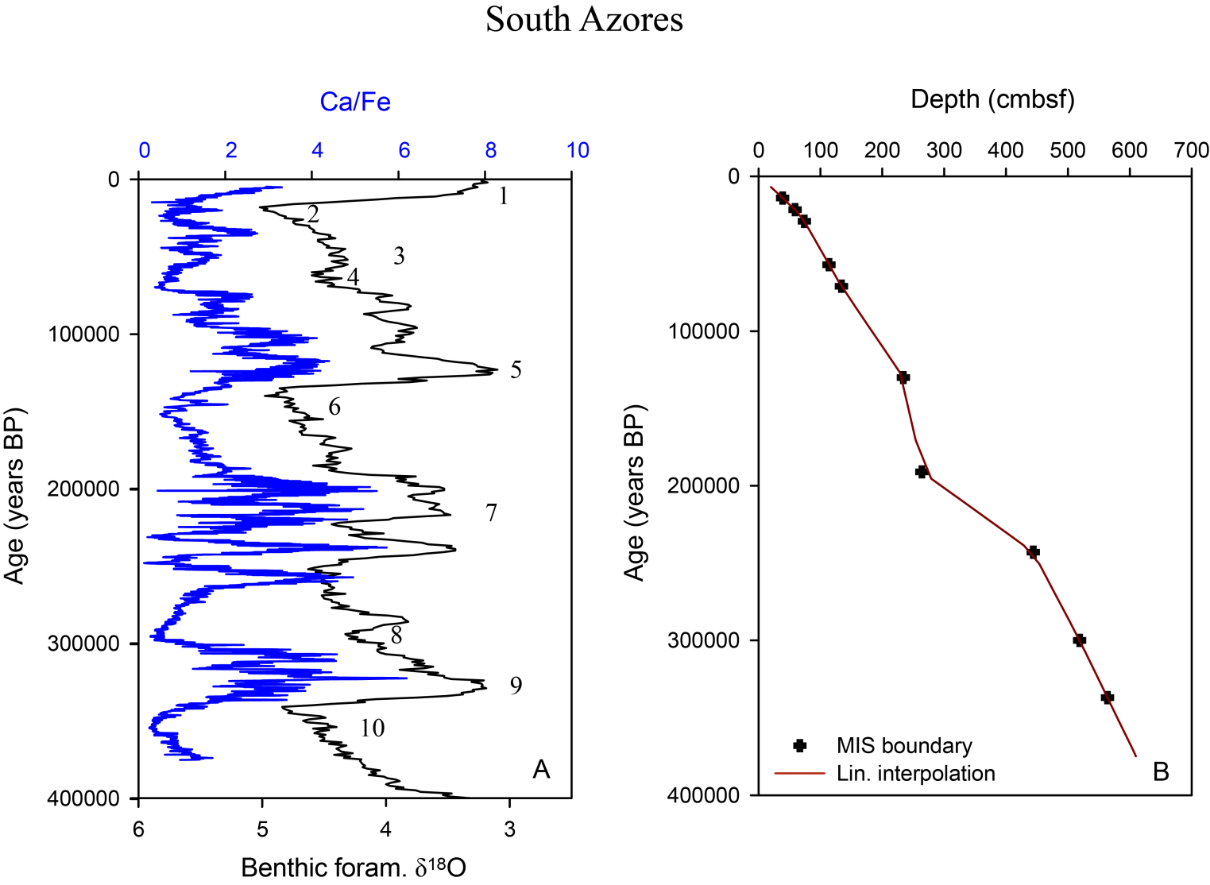


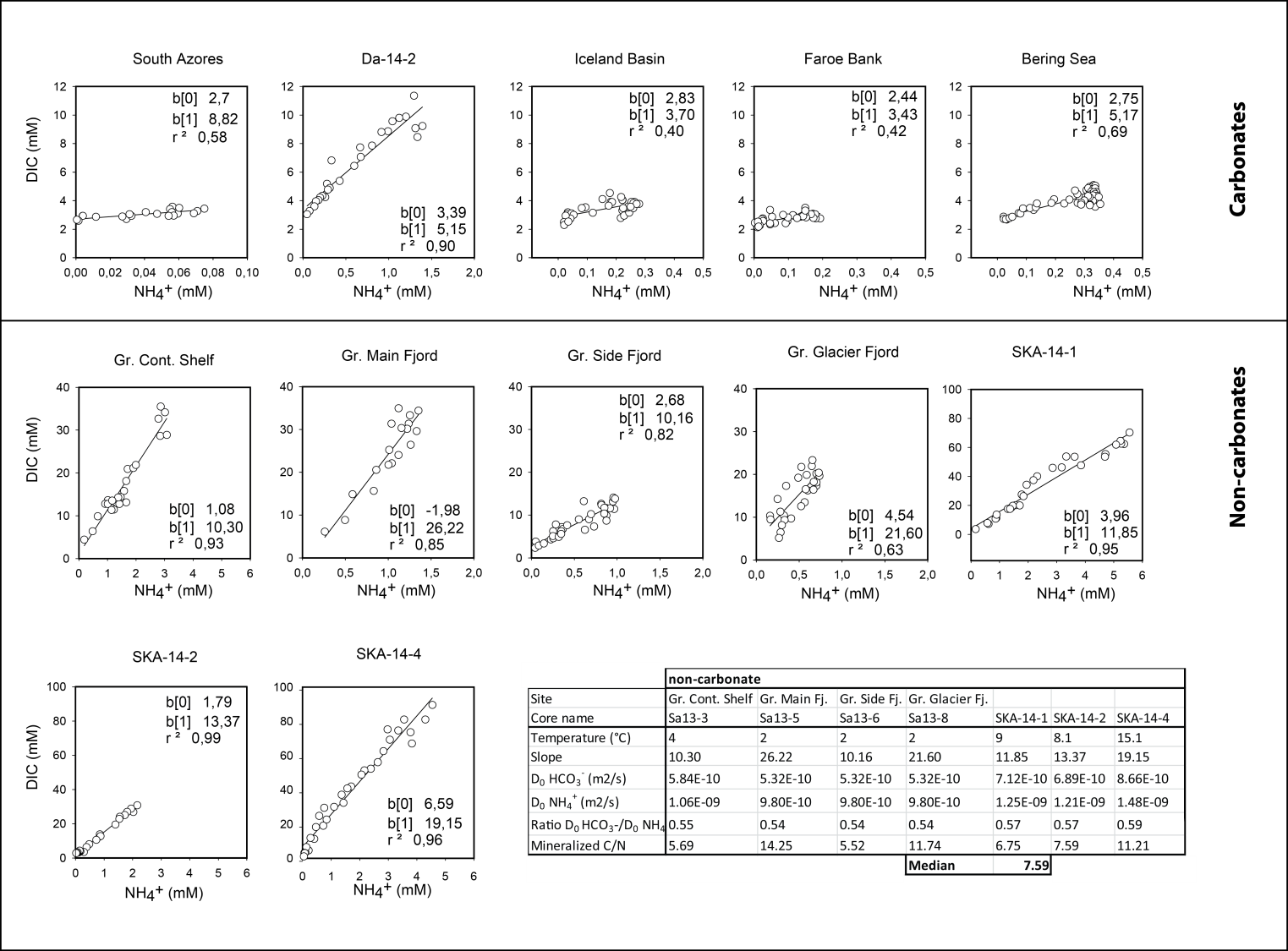
**Suppl. Figure S4**: Parameter plots used to calculate mineralized C:N ratios. A linear regression was done between DIC and NH_4_+; b[1] is the slope of the fit and b[0] the intercept of the fit. Additional sites from expedition SKA-14 (Skagerrak, Baltic Sea) were added to increase the number of sites. We used only non-carbonated sediments to determine the DIC:NH_4_+ ratio. At the bottom of the figure, the table provides the diffusion coefficients corrected for temperature and salinity involved in the determination of the mineralized C:N (see methods). The median C:N ratio was 7.6, and this value was used to convert ammonium production rates into carbon oxidation rates.

**Suppl. Table S1**: Data input radiocarbon age (^14^C) with modelled and calibrated age. Data fom the Iceland basin have previously been published in (Van Nieuwenhove et al., 2018). Data from the Greenlandic fjords have previously been published in (Pelikan et al., 2019).

|  |  |  |  |  |  | Unmodelled (BP) | | Unmodelled (BP) | |  |  |  |  |
| --- | --- | --- | --- | --- | --- | --- | --- | --- | --- | --- | --- | --- | --- |
|  |  |  |  |  |  | 1 sigma range | | 2 sigma range | |  |  |  |  |
| **Site** | **core name** | **Labcode samples** | **Depth (average) cmbsf** | **14C Age** | **Error** | **from** | **to** | **from** | **to** | **μ** | **σ** | **Mean Modelled Age (yr cal. BP)** | **Error** |
| Faroe Bank (GC03) | Da-12-2-GC03 | AAR-21487 | 10.5 | 7235 | 45 | 7751 | 7645 | 7806 | 7597 | 7699 | 53 | 7680 | 50 |
|  |  | AAR-21488 | 105.5 | 15850 | 60 | 18782 | 18645 | 18840 | 18569 | 18709 | 68 | 18647 | 69 |
|  |  | AAR-21489 | 130.5 | 16780 | 75 | 19880 | 19633 | 20000 | 19550 | 19769 | 118 | 19787 | 107 |
|  |  | AAR-21491 | 210.5 | 20090 | 110 | 23871 | 23562 | 24021 | 23406 | 23712 | 156 | 23771 | 138 |
|  |  | AAR-21493 | 275.5 | 23480 | 150 | 27525 | 27256 | 27646 | 27105 | 27380 | 137 | 27456 | 122 |
|  |  | AAR-21494 | 380.5 | 31400 | 350 | 35270 | 34575 | 35676 | 34247 | 34953 | 355 | 35426 | 340 |
|  |  | AAR-21495 | 430.5 | 35850 | 600 | 40748 | 39386 | 41339 | 38803 | 40074 | 654 | 40474 | 484 |
|  |  | AAR-21496 | 475.5 | 45200 | 1900 | 49611 | 46826 | ... | 45602 | 47836 | 1281 | 45486 | 721 |
|  |  | AAR-21492 (outlier) | 255.5 | 23100 | 140 | 27265 | 26847 | 27370 | 26604 | 27014 | 202 |  |  |
|  |  | AAR-21490 (outlier) | 180.5 | 20240 | 100 | 24020 | 23730 | 24154 | 23580 | 23871 | 144 |  |  |
| Iceland Basin (GC01) | Da-12-11-GC01 | AAR_17560 | 21.5 | 598 | 25 | 288 | 150 | 295 | 142 | 235 | 43 | 273 | 27 |
|  |  | AAR_23190 | 45.5 | 1416 | 25 | 987 | 920 | 1038 | 905 | 963 | 35 | 946 | 30 |
|  |  | AAR-23191 | 85.5 | 2234 | 25 | 1879 | 1801 | 1920 | 1747 | 1837 | 40 | 1853 | 38 |
|  |  | AAR-23192 | 140.5 | 3819 | 26 | 3817 | 3719 | 3860 | 3675 | 3766 | 48 | 3739 | 46 |
|  |  | AAR_17561 | 188.5 | 4562 | 35 | 4828 | 4730 | 4845 | 4682 | 4775 | 44 | 4802 | 29 |
|  |  | AAR-24460 | 235 | 7080 | 35 | 7595 | 7517 | 7637 | 7482 | 7558 | 38 | 7567 | 37 |
|  |  | AAR-24461 | 280 | 8383 | 35 | 9025 | 8930 | 9097 | 8849 | 8974 | 58 | 8981 | 44 |
|  |  | AAR_17562 | 330.5 | 9114 | 30 | 9916 | 9764 | 10026 | 9692 | 9852 | 81 | 9910 | 72 |
|  |  | AAR-24462 | 370 | 9583 | 47 | 10537 | 10393 | 10583 | 10286 | 10451 | 74 | 10503 | 59 |
|  |  | AAR-24463 | 410 | 10484 | 42 | 11809 | 11474 | 11895 | 11360 | 11640 | 143 | 11573 | 129 |
| Gr. Continental Shelf (20G) | SA13-ST3-20G | AAR2067 | 163.5 | 2362 | 25 |  |  |  |  |  |  | 2048 | 43 |
|  |  | AAR2068 | 346 | 6446 | 28 |  |  |  |  |  |  | 6940 | 49 |
|  |  | AAR2069 | 435 | 9033 | 39 |  |  |  |  |  |  | 9617 | 55 |
|  |  | AAR2070 | 514 | 9896 | 39 |  |  |  |  |  |  | 10921 | 79 |
| Gr. Side Fjord (40G) | SA13-ST6-40G | AAR21687 | 50 | 1372 | 24 |  |  |  |  |  |  | 919 | 32 |
|  |  | AAR21690 | 173.5 | 2668 | 2 |  |  |  |  |  |  | 2362 | 43 |
|  |  | AAR21691 | 196 | 3356 | 30 |  |  |  |  |  |  | 3218 | 54 |
|  |  | AAR21692 | 260 | 4458 | 40 |  |  |  |  |  |  | 4647 | 75 |
| Gr. Main Fjord (30G) | SA13-ST5-30G | AAR20956 | 539 | 815 | 25 |  |  |  |  |  |  | 457 | 26 |
|  |  | AAR20955 (outlier) | 25 | 652 | 25 |  |  |  |  |  |  |  |  |

**Suppl. Table S2**: Parameters used to calculate NH_4_^+^ production rates in PROFILE.

| **Parameters** | **Faroe Bank** | **Iceland Basin** | **Greenland Cont. Shelf** | **South Azores** | **Eastern Equ. Pacific** | **Bering Sea** |
| --- | --- | --- | --- | --- | --- | --- |
| **Site** | Da-12-2 | Da-12-1 | Sa-13-3 | Da-14-1 | Leg201-1226B | Leg323-U1342-B |
| Temperature (°C) | 8 | 3 | 4 | 3.2 | 1.8 to 25.6 | 2 to 6 |
| Depth at top of calculation domain (mbsf) | 0.12 | 0.12 | 0.0125 | 0.95* | 1.3 | 0.54 |
| Depth at bottom of calculation domain (mbsf) | 5.925 | 4.215 | 6.05 | 6.04 | 417.7 | 42.57 |
| Max number of equally spaced zones in interpretation | 12 | | | | | |
| Type of boundary conditions (C= concentration) | Top: C, Bottom: C | | | | | |
| Top boundary condition (mol m^-3^) | 0.010435179 | 2.61E-02 | 1.50E-01 | 9.69E-04* | 5.17E-02 | 2.20E-02 |
| Bottom boundary condition (mol m^-3^) | 0.192823958 | 2.16E-01 | 3.09E+00 | 7.00E-02 | 1.65E-01 | 2.88E-01 |
| Molecular diffusion coefficient in water (D_0_, m^2^ s^-1^) | 1.21E-09 | 1.02085E-09 | 1.06E-09 | 1.03E-09 | modified #^¶^ | modified #^¶^ |
| Expression for sediment diffusivity (Ds, m^2^ s^-1^) | Ds= D_0_ /(1+3*(1- φ)) | | | | Ds= D_0_/ (τ2) | Ds= D_0_ /(1+3*(1- φ)) |
| Concentration in water column (C0) (mmol L^-1^) | 0 | | | | | |
| Minimum for production/consumption rate (mol m^-3^ s^-1^) | 0 | | | | | |
| Maximum for production/consumption rate (mol m^-3^ s^-1^) | 1.00E+20 | | | | | |
| Maximum deviation (in %) when accepting a calculated minimum | 0.001 | | | | | |
| Level of significance in the F statistics | 0.05 | | | | | |
| Points excluded | 4 (0.3225, 4.75, 5.125, 5.525mbsf) | 1 (1.415mbsf) | 0 | Removed  negative values | 0 | 2 (0.11 and 0.30 mbsf) |
| Number of first zones proposed by PROFILE | none | 2 | 7 | none | 6 | 4 |
|  |  |  |  |  |  |  |
| Final number of zones | 3 | 3 | 3 | 1 | 3 | 3 |
|  |  |  |  |  |  |  |
| Corrected the number of zones proposed by PROFILE | yes | yes | yes | yes | no | yes |
| *#The molecular diffusion coefficient in Eastern Equatorial Pacific and Bering Sea was calculated according to the temperature gradient (Equations 8 and 9.). * the upper boundary conditions were set at 0.95 mbsf in the South Azores as the NH_4_^+^ concentration were below the limit od quantification within the upper 0.95mbsf.* | | | | | | |
